# Connexins in the Heart: Regulation, Function and Involvement in Cardiac Disease

**DOI:** 10.3390/ijms22094413

**Published:** 2021-04-23

**Authors:** Antonio Rodríguez-Sinovas, Jose Antonio Sánchez, Laura Valls-Lacalle, Marta Consegal, Ignacio Ferreira-González

**Affiliations:** 1 Cardiovascular Diseases Research Group, Department of Cardiology, Vall d’Hebron Institut de Recerca (VHIR), Vall d’Hebron Barcelona Hospital Campus, Vall d’Hebron Hospital Universitari, Passeig Vall d’Hebron 119-129, 08035 Barcelona, Spain; joseant.msq@gmail.com (J.A.S.); lvalls@ibecbarcelona.eu (L.V.-L.); marta.consegal@vhir.org (M.C.); 2 Departament de Medicina, Universitat Autònoma de Barcelona, 08193 Bellaterra, Spain; 3 Centro de Investigación Biomédica en Red sobre Enfermedades Cardiovasculares (CIBERCV), Instituto de Salud Carlos III, 28029 Madrid, Spain; 4 Centro de Investigación Biomédica en Red (CIBER) de Epidemiología y Salud Pública (CIBERESP), Instituto de Salud Carlos III, 28029 Madrid, Spain

**Keywords:** connexin, Cx43, gap junction, hemichannel, mitochondria, nucleus, heart, cardiomyocyte

## Abstract

Connexins are a family of transmembrane proteins that play a key role in cardiac physiology. Gap junctional channels put into contact the cytoplasms of connected cardiomyocytes, allowing the existence of electrical coupling. However, in addition to this fundamental role, connexins are also involved in cardiomyocyte death and survival. Thus, chemical coupling through gap junctions plays a key role in the spreading of injury between connected cells. Moreover, in addition to their involvement in cell-to-cell communication, mounting evidence indicates that connexins have additional gap junction-independent functions. Opening of unopposed hemichannels, located at the lateral surface of cardiomyocytes, may compromise cell homeostasis and may be involved in ischemia/reperfusion injury. In addition, connexins located at non-canonical cell structures, including mitochondria and the nucleus, have been demonstrated to be involved in cardioprotection and in regulation of cell growth and differentiation. In this review, we will provide, first, an overview on connexin biology, including their synthesis and degradation, their regulation and their interactions. Then, we will conduct an in-depth examination of the role of connexins in cardiac pathophysiology, including new findings regarding their involvement in myocardial ischemia/reperfusion injury, cardiac fibrosis, gene transcription or signaling regulation.

## 1. Introduction

**Figure 1:**
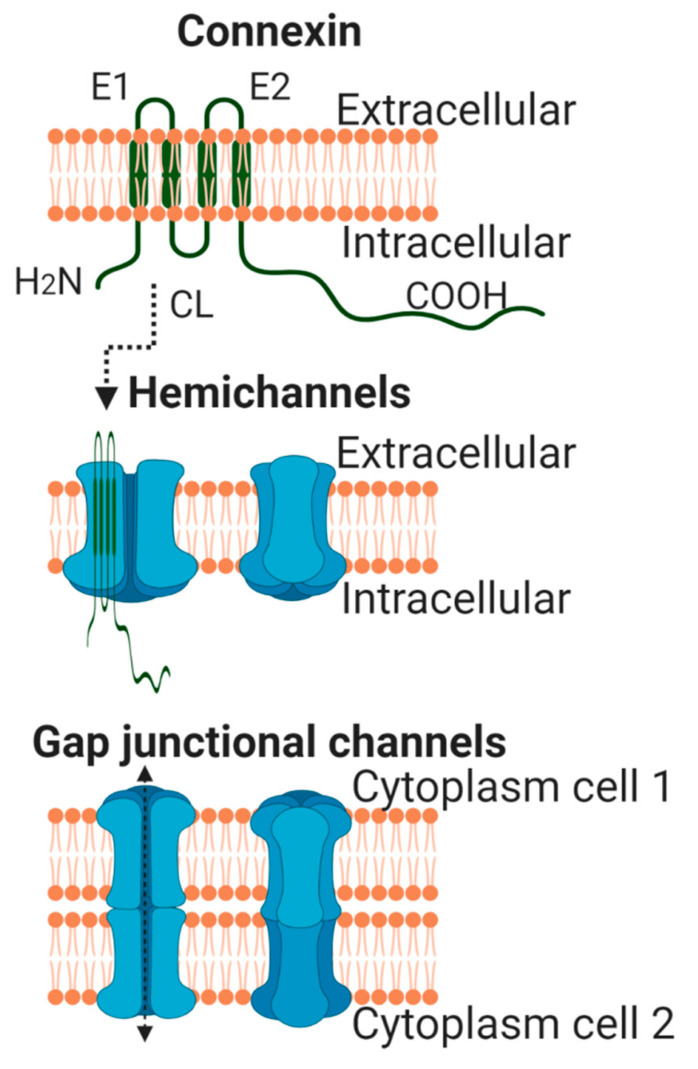
Schematic view of a single connexin molecule located at the plasma membrane (upper panel). Lower panels depict hemichannels and intercellular or gap junctional channels. Created with Biorender.com (accessed on 15 April 2021). Modified from [11,12].

Connexins are a large family of highly homologous transmembrane proteins, comprising 20 and 21 different isoforms in mice and humans, respectively [1,2,3]. All these isoforms are named according to their expected molecular weight in kDa and have distinct biophysical properties. However, and despite their electrophysiological differences, all connexins share a similar structure. They are oriented so that the amino- and carboxyterminal (CT) tails of the protein are located within the cell cytoplasm, and they include four transmembrane-spanning α-helix domains linked by two extracellular segments (E1 and E2) and one cytoplasmic loop (Figure 1) [4]. Differences between connexin isoforms are mostly due to variations in the amino acid sequence at the CT domain, although also, to some extent, at the cytoplasmic loop [5]. The CT domain has multiple serine, threonine and tyrosine residues susceptible to phosphorylation, which is a very important phenomenon regulating connexin trafficking, assembly and function [6,7,8,9], and is largely responsible for the appearance of multiple bands in Western blots. Connexin 43 (Cx43) is, by far, the best-known and most ubiquitously expressed isoform, and it is encoded by the GJA1 gene, located, in humans, in chromosome 6 [10]. The gene nomenclature for the two other most common human cardiac connexin isoforms, Cx40 and Cx45, is GJA5 and GJC1, respectively [2].

**Figure 2:**
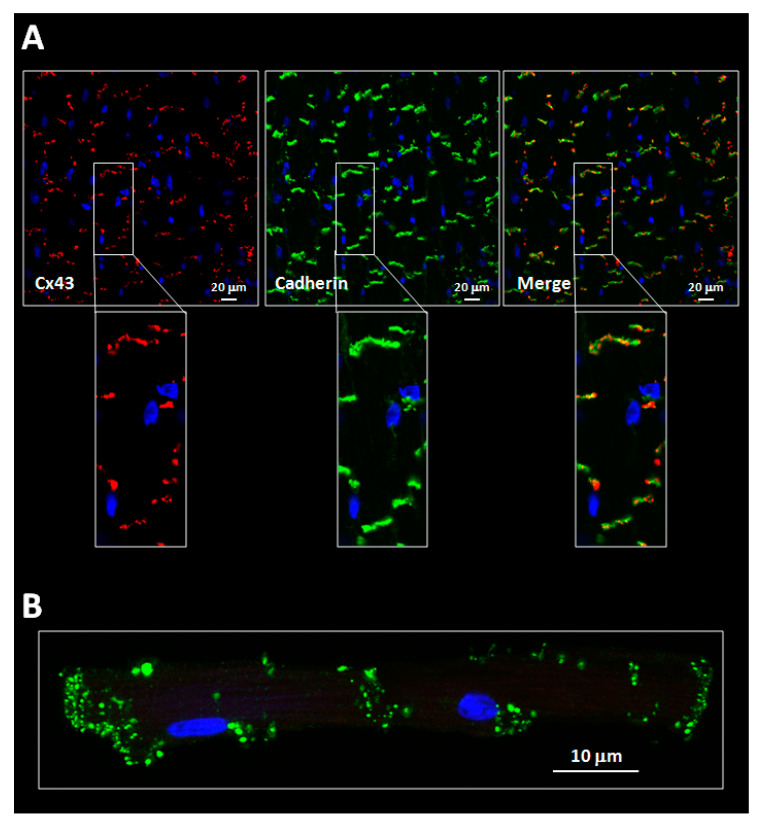
(**A**) Confocal images showing expression of connexin 43 (Cx43, red) in cardiac slices obtained from wild-type mice hearts. Cx43 is mostly expressed at the cell poles, within the intercalated discs, where it colocalizes with pan-cadherin (green) (see magnified images). (**B**) Confocal image of a pair of end-to-end connected mice cardiomyocytes showing Cx43 expression (green) at gap junctions. Nuclei were stained with Hoechst 33642 (blue).

All connexin isoforms are integral components of plasma membranes where they form channels around a central pore. These channels are formed by the oligomerization of six individual connexin molecules and are known as hemichannels or connexons (Figure 1) [4,13]. Hundreds to thousands of these hemichannels gather in plaques, termed gap junctions, where they dock with opposing connexons from adjacent cells, forming intercellular channels (Figure 1) [4,13]. In cardiomyocytes, gap junctions are mainly located at the cell poles (Figure 2), perpendicular to the long axis of the cell, within the intercalated discs, which are complex structures in which plasma membranes of neighboring cells are in close contact and that also include adherens junctions, hemichannels and ion channels [14,15]. Three cysteine residues, located in each extracellular loop of each connexin molecule, are important in the docking process. Disulfide bonds between these cysteines are needed to create the β-sheet conformation required for the interaction between the two opposing hemichannels [4,5,16]. In fact, Cx43 lacking these cysteines is not able to form gap junctional intercellular channels [17]. Connexons can be formed by a unique connexin isoform or by several connexin isoforms (giving rise to homomeric or heteromeric connexons, respectively), although not all are compatible [16]. In turn, intercellular channels can be homotypic (formed by connexons with the same composition) or heterotypic (when each connexon has a different composition) [18,19]. Such mixed conformations modify channel electrophysiological properties and alter gap junctional conductance [20,21].

Intercellular channels put into contact the cytoplasms of connected cells, allowing the transfer of ions and small intracellular molecules between them, in a process known as gap junctional intercellular communication (GJIC) [16]. GJIC is a conserved phenomena in many phyla, from invertebrates to chordates [3,16]. This fact gives an idea of the critical importance of GJIC to coordinate cell responses and function in multicellular organisms. This is especially important in the heart, where gap junctions and GJIC are responsible for maintaining a coordinated cardiac contraction.

In addition to their location at the plasma membrane, some connexins have been described in other less conventional cell structures, including the nucleus [22] and the mitochondria [23,24,25]. In this review, we will, first, provide an overview on general aspects of connexin biology, including synthesis and degradation, regulation and interactions, with especial interest in those isoforms expressed in the heart, followed by an in-depth examination of their involvement in cardiac pathophysiology. Finally, we will highlight new findings regarding their implication in some non-canonical functions, including cardiac fibrosis, gene transcription and signaling regulation, and in cardioprotection, which are probably related to their presence at these newly described locations.

## 2. General Aspects of Connexin Biology

### 2.1. Synthesis and Degradation of Connexins

**Figure 3:**
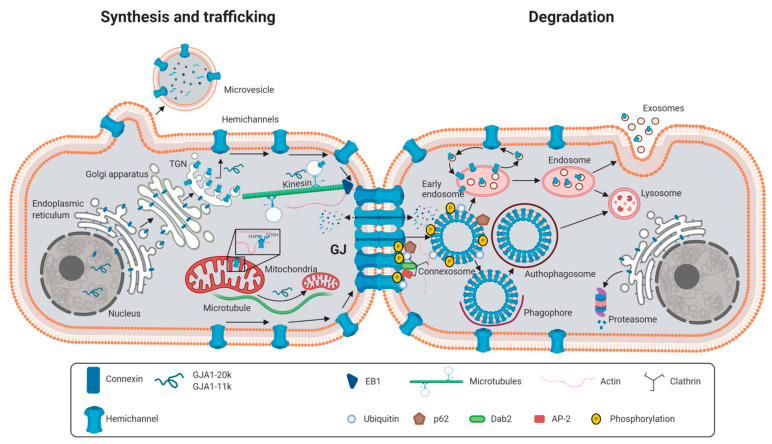
Synthesis and intracellular trafficking (left cell) and degradation (right cell) of connexins. Connexins are synthesized at the endoplasmic reticulum but are oligomerized into hemichannels at the cis-Golgi apparatus or at the trans-Golgi network (TGN). Transport to the plasma membrane occurs predominantly through microtubules and motor proteins such as kinesin, but actin may also play an important role. Direct delivery of vesicles containing hemichannels may also occur. Cx43 reaches mitochondria using the TOM/TIM system of protein import, in an Hsp90-dependent manner. N-terminally truncated isoforms can be found at the cytoplasm and nucleus. Degradation occurs through formation a double-membrane structure, termed connexosome. Connexosomes can then be targeted to the endolysosomal or the autophagosomal pathways. Connexins can also be degraded after synthesis at the endoplasmic reticulum by the proteasome. Release of microvesicles and exosomes containing connexins is also depicted. GJ indicates gap junctions. Created with Biorender.com (accessed on 15 April 2021). Modified from [8,12,27].

Synthesis of connexins takes place at the endoplasmic reticulum, where folding and post-translational modifications occur (Figure 3) [12,26,27]. However, oligomerization into hemichannels depends on the specific connexin isoform. Oligomerization into hexamers takes place, for the main cardiac isoform, Cx43, and probably also for Cx40, at the trans-Golgi network [26,27,28]. While progressing from the cis-Golgi to the trans-Golgi network, connexins are submitted to further sorting and post-translational modifications [27]. Connexons are then transported in 100- to 150-nanometer vesicles to the cell membrane, as demonstrated by live cell imaging of fluorescent-tagged Cx43, in a process in which microtubules, but also actin filaments, are essential [29,30]. Some evidence indicates that, in this way, hemichannels might be directly delivered to specific subdomains of the cell membrane, hosting anchor proteins (i.e., adherens junctions), near cell-to-cell junctions [14], in a process termed targeted delivery [27]. The minus end of microtubules colocalizes with the trans-Golgi network, where the cargo proteins are loaded onto microtubule molecular motors, including kinesin, and delivered to the cell periphery (Figure 3) [31]. Microtubule highways then reach and anchor to adherens junction complexes, including proteins such as N-cadherin or β-catenin, within the plasma membrane, delivering Cx43-containing vesicles to the cell border at the intercalated disc [27]. In this process, the microtubule plus-end-binding protein EB1, which specifically interacts with either β-catenin molecules bound to N-cadherin at adherens junctions [14], or with desmoplakin at desmosomes [32], both located in close proximity to gap junctions, is important. Vesicles transported along microtubules travel to the cell periphery at a fast rate of about 1 µm/s [14]. In contrast to microtubules, the role of actin filaments in vesicle transport is less clear, and it has been suggested that they may constitute a pause station as a reserve of hemichannels waiting to be delivered to the membrane when needed [27].

Targeted delivery, thus, allows connexons to be incorporated at the periphery of existing gap junctions [33,34]. Indeed, connexons are found at high concentrations in areas surrounding gap junctions, in a region termed the perinexus [35,36,37]. From these areas, connexons are incorporated, by lateral diffusion, into pre-existing gap junctions, in a process regulated by zonula occludens-1 [35,36,37]. These connexin-rich areas constitute regions where connexin can interact with intracellular signaling pathways, kinases, the cytoskeleton and other junctional proteins [35,36,38,39]. In addition, diffusion may also exist in the case of free, unopposed hemichannels delivered to non-junctional membranes [27].

On the other hand, nascent plaques are formed by the gathering of connexons in concrete regions within the plasma membrane, termed formation plaques [40,41]. In them, the intermembrane distance between cells is similar to that found in mature gap junctions, as demonstrated by freeze-fracture electron microscopy analysis [40,41]. These areas would, presumably, provide hemichannels the opportunity to dock to form intercellular channels [42]. These small aggregates of channels will grow over time, fusing, and forming a densely packed gap junction [42]. Docking of hemichannels occurs only when the distance between adherent cells is about 1/5 of the normal space [43]. Importantly, a critical determinant of channel opening is the gap junction size, and it has been estimated that a minimum of 200–300 channels are required for it [44]. However, even in large plaques, only a small fraction of channels (between 10 and 20%) are usually functional [44]. Older hemichannels are, then, removed from the plaque center in pleiomorphic vesicles of different sizes [33,34].

Connexins are submitted to a rapid turnover, with some isoforms, including Cx43, having very short half lives (1.5 to 5 h) [45,46,47]. Initial studies demonstrated that ubiquitinination typically signaled for connexin degradation [48], a phenomenon that would rapidly modify the number of gap junctional channels present at the cell membrane [49,50]. However, some differences in the mechanism of degradation seem to exist, depending on the exact location of connexins. Thus, misfolded connexins, together with hemichannels in their transit to the plasma membrane, can be mainly degraded through the 26S proteasome (Figure 3) [50], in a process that can be both ubiquitin-dependent or independent. The role of the ubiquitin/proteasome system in connexin degradation was first reported for Cx43 [51], as treatment with proteasome inhibitors enhanced Cx43 levels and reduced degradation rates [51,52]. Both Cx43 and Cx32 have been shown to undergo endoplasmic reticulum-associated proteasome-mediated degradation (ERAD) during or after protein synthesis [53]. Dislocation of Cx43 from the endoplasmic reticulum seems to be necessary for ERAD, a process in which CIP75 (Cx43-interacting protein of 75 kDa) plays a key role [54]. In addition, CIP75 mediates the interaction between Cx43 and the proteasome [54]. It has been suggested that up to 40% of newly synthetized Cx43 can undergo ERAD, a process regulated by cytosolic stress [55]. The reasons for such a high rate of connexin degradation are unknown.

On the other hand, degradation of connexins that form part of mature gap junctions has been described to occur through two different pathways, the endolysosomal pathway and the autophagosomal pathway (Figure 3) [8,48,50,56,57,58]. The first steps in these two processes seem to be common and may require connexin ubiquitination through E3 ubiquitin ligases [50]. Once ubiquitinated, Cx43 interacts with the ubiquitin-binding protein Eps15 at the plasma membrane, followed by recruitment of clathrin, the GTPase dynamin, the clathrin adaptors AP-2 and DAB2 and the protein p62, which together target ubiquitinated Cx43 to the endocytic pathway [50,59,60]. This process ends with the formation of a double-membrane structure, termed annular gap junction or connexosome, containing connexins from both cells [49,50,61,62]. At this stage, connexins might be recycled and transported again to the plasma membrane [49]. If this is not the case, and following endocytosis, internalized annular gap junctions are, then, trafficked inward by myosin VI and targeted for degradation [50]. According to this hypothesis, at this point, the endolysosomal and autophagosomal pathways would diverge.

Under some conditions, early endosomes transport ubiquitinated Cx43 to lysosomes, a process in which the ubiquitin-binding proteins Hrs and Tsg101 act as critical regulators [48,50]. Binding of both proteins to the internalized gap junction occurs, for example, after 12-O-tetradecanoylphorphol 13-acetate (TPA) or epidermal growth factor (EGF) treatment [50], which induced further phosphorylation and ubiquitination of the protein [56,57]. In fact, siRNA-mediated knock down of Tsg101 in HM embryonic stem cells led to increased Cx43 and Cx45 levels, a prolonged half-life of both proteins and increased dye transfer between connected cells [63]. Interaction between Tsg101 and connexins seems to occur at the CT domain of the protein [63]. Then, fusion of the endosome to the outer non-junctional membrane of the annular gap junction occurs, followed by binding of a lysosome to the endosome, thus driving gap junction degradation [50].

The second pathway, the autophagosomal or phago-/lysosomal pathway, occurs in untreated or starved cells and in diseased tissue [50]. Under these conditions, reduced phosphorylation and ubiquitination of Cx43 target annular gap junctions to the autophagosomal degradation pathway [50,60]. Indeed, interruption of macroautophagy has been shown to result in retention of Cx43 at the plasma membrane, an effect associated with enhanced gap junction-mediated intercellular dye diffusion [60]. In this process, p62 drives the annular gap junction to a forming autophagosome that would, then, fuse with a lysosome to degrade connexins [50].

In some cells, the 26S proteasome can also play a role in gap junction degradation. Indeed, treatment with proteasome inhibitors has been shown to increase the size of Cx43 gap junctional plaques [46,64]. However, the involvement of the proteasome is probably indirect [50]. Some data seem to suggest that it is not the direct ubiquitination of Cx43 but instead that of Akt which signals for its degradation [65]. According to this hypothesis, Akt phosphorylation of Cx43 would promote stabilization of gap junctions, whereas Akt ubiquitination would remove this stabilizing factor [65].

### 2.2. Permeability and Conductance of Connexin Channels

The biophysical properties of connexin channels are complex, include voltage and chemical gating, permeant selectivity and rectification [16,66,67,68,69,70,71,72] and depend on whether they are unopposed hemichannels or docked to an opposing connexon to form an intercellular channel [72,73]. GJIC is determined by several factors, including the number of available channels, single-channel conductance and permeability and the open probability of each channel [5]. The pore diameter ranges between 6.5 and 15 Å [16,74,75], depending on the specific connexin isoform, and is wide enough to be permeable to water, most ions, including Cl^−^, Na^+^, K^+^ and Ca^2+^, most cytosolic second messengers, such as IP3, adenosine, ADP, ATP, cAMP, cGMP, polypeptides, microRNAs and small interfering RNAs [76,77,78,79,80,81], and even glucose [82]. Gap junctional channels behave rather as unselective pores for small molecules, whereas selectivity becomes more apparent as the size of the molecule increases, which causes differences in permeability depending on the connexin isoform [16,76,77,78,79,80,81]. However, gap junctional permeability depends not only on size but also on the net charge of the molecule, as it has been demonstrated with the use of different fluorescent probes of similar molecular weight but opposite charge [79]. As an example, Cx43-formed gap junctional channels show a higher pore size than those formed by Cx32 [16], but they are more cation-selective, with differences in permeability to adenosine, ATP, ADP and AMP [83].

Similar to chemical permeability, maximal unitary conductance depends on the specific connexin isoform. Maximal unitary conductance of junctional channels ranges from about 5–15 for Cx36 to about 310 pS for Cx37, with values for cardiac connexins being 32, 110 or 175 pS for Cx45, Cx43 or Cx40, respectively [16,84,85]. Moreover, the maximal unitary conductance of free, undocked hemichannels is about twice that of the corresponding gap junction channels (i.e., about 62 or 220 pS for Cx45 or Cx43) [86]. Two different gating mechanisms have been proposed [16,66,68,69,71] that carry the open state to partially or fully closed states. The first gating mechanism is a fast, voltage-driven system that occurs within a few milliseconds and that changes channel conformation from the fully open state to an almost completely, but not fully, closed state (voltage gating), and vice versa. In this case, gap junctional conductance reaches about 5 to 30% of maximal conductance and creates what is known as a residual state or substate [16,70,71]. The second gating mechanism is a slower system (up to 30 ms) that brings the channel to complete closure in response to chemical interactions (chemical gating) or to changes in voltage (loop gating).

Both the unitary conductance (γ_j_) of junctional channels and their open probability (i.e., the number of open junctional channels, P_0_), together with the total number of channels (N), determine macroscopic gap junctional conductivity (G_j_ = N × γ_j_ × P_0_) [5,16,87]. In this regard, it is important to consider that regulation of gap junction channels may have opposite effects in different variables [84,88]. This would be the case of the protein kinase C (PKC) activator TPA, which induces an increase in macroscopic conductivity in pairs of neonatal rat cardiomyocytes, despite causing a decrease in unitary conductance and permeability, with the former being explained by an enhanced open probability of Cx43-formed channels [89]. Moreover, it has been demonstrated that the residual state of Cx43 is more anion-selective and allows electrical cell-to-cell communication, but it limits metabolic coupling [70]. Thus, any condition modifying gap junction regulation should be explained in terms of its consequences on permeability, selectivity and electrical conductance, but not simply as an increase or decrease in GJIC [84].

### 2.3. Gating Regulation of Connexin Channels

A variety of physical/chemical stimuli that are known to play an important role in cardiac pathophysiology, including transmembrane and transjunctional voltages (Vm and Vj, respectively), pH and intracellular Ca^2+^ concentrations, are involved in regulation of connexin channel gating and are discussed below.

#### 2.3.1. Gating by Transjunctional Voltage (Vj) and Transmembrane Potential (Vm)

The conductance of most intercellular connexin channels is sensitive to Vj, while only few connexin isoforms are sensitive to both Vj and Vm. The exact molecular structures responsible for both Vj and Vm gating events have not been completely identified. However, the fact that most connexins are only sensitive to Vj, but not to Vm, may indicate that the voltage sensor must reside within the channel pore. Only at this location would it be able to sense the voltage drop along the length of the pore, but not changes in Vm [90]. Despite this hypothesis, other parts of the molecule, depending on the connexin isoform, have been proposed to also be involved in Vj gating, including the aminoterminal (NT) domain, the first transmembrane segment, the cytoplasmic loop and the carboxyterminal (CT) domain [13]. Truncation or modifications of the CT domain in some connexins, such as Cx43, have been shown to eliminate Vj gating transitions to the residual state [69,91,92,93], leading to the proposal that fast Vj gating follows a particle-receptor model (or “ball-and-chain” model), in which the long, flexible CT domain binds to a specific region within the cytoplasmic loop, which would reside in the pore vestibule, to partially occlude the channel [69,91].

In fact, each hemichannel within an intercellular channel may contain two different Vj gates, responsible for the fast and slow gating mechanisms previously mentioned [91]. Fast Vj gating events correspond to fast transitions between the open state and one or more substates [90] and is a connexin-specific phenomenon, with Cx26, Cx30, Cx37, Cx40, Cx46 and Cx50 closing under positive voltages at their cytoplasmic face, whereas Cx31, Cx31.9 (mCx30.2), Cx32, Cx43, Cx45 and Cx57 close under negative potentials [13,91]. In contrast, slow or loop gating corresponds to a series of small amplitude transitions between multiple intermediate states that together give rise to an event with a slow time course that fully closes the channel [90]. In all connexin channels examined to date, loop gating is favored at inside negative voltages [90]. Furthermore, studies in cell lines expressing Cx43 suggested that both Vj gates operate in series [94]. Thus, although single-channel records conducted at low voltages (Vj < 60 mV) have demonstrated transitions from the main open state to both the residual and closed states, transitions between the main open state and the residual state are mainly observed at moderate Vj gradients (60–100 mV), while transitions between the substate and the fully closed state occur at a larger Vj [94,95]. Importantly, truncation of the CT domain enables the two Vj gating mechanisms to be dissociated, providing evidence that the fast and slow gates constitute separate mechanisms dependent on different structures within the molecule [69,92].

On the other hand, a third gate, sensible to changes in Vm, regulates channel transitions into and out of the fully closed state in some connexin isoforms, including Cx43 [91]. Previous studies demonstrated that an equal depolarization of both connected cells decreases Cx43 gap junctional conductivity [96]. The Vj and Vm mechanisms of voltage gating might be dependent on different structural areas within the connexin molecule, based on the striking differences, for a given connexin, in the polarity of closure, voltage sensitivity and kinetics [91]. Indeed, clusters of charged residues within separate domains of the connexin molecule have been identified as integral parts of the Vj and Vm sensors [91]. Mutational studies of Cx43 have suggested that the Vm sensor is located in a different region of the CT domain than that involved in fast Vj gating [96]. However, interactions between both gating sensors cannot be excluded [96].

Under physiological conditions, it is difficult to attain the voltage gradients required to close most connexin channels, although there are some exceptions. Vj gating may occur in the case of Cx45, the most sensitive connexin isoform. Vj gating in this isoform would prevent retrograde impulse propagation from the working myocardium, expressing mainly Cx43, to the conduction system [13]. On the other hand, gating regulation by Vj and Vm of Cx43-formed gap junctions can be important in cardiac pathophysiology. Changes in both Vj and Vm may occur under some pathological situations, such as during myocardial ischemia, especially at the border zone surrounding the ischemic region. Hypothetically, these changes may help to close gap junctional channels, thus electrically isolating the area at risk from the remaining myocardium. This would prevent the spreading of arrhythmogenic events, such as enhanced abnormal automaticity or triggered activity due to afterdepolarizations, appearing within the ischemic myocardium. On the other hand, however, electrical isolation may lead to the appearance of areas of conduction block that might drive reentry. Furthermore, uncoupling by membrane depolarization of Cx43 channels (i.e., Vm gating) might constitute a protective mechanism to prevent electrotonic influences of pathologically depolarized cells [13].

#### 2.3.2. Gating by Intracellular pH

Modulation of gap junctional conductance by intracellular pH is a well-known feature of connexin channels [4,13,85,97]. Initial studies conducted in the 1970s and early 1980s demonstrated an association between a reduction in intracellular pH and an increase in junctional resistance [98,99,100]. Later on, studies in Xenopus oocytes expressing different connexin isoforms confirmed that cytoplasm acidification reduces gap junctional intercellular communication in most, if not all, isoforms [13,101,102,103]. However, pH gating of connexin channels (i.e., half maximal uncoupling and the number of channels active at physiological pH) depends on the specific connexin isoform [13,101,102]. While channels formed by Cx43 are mainly in the open configuration at pH 7.2 [103] and a slight reduction in intracellular pH is able to partially close them, a higher intracellular acidification is needed to achieve the same degree of gap junction closure for Cx32 channels [101,102]. Dependence on intracellular pH has been analyzed for a wide variety of connexin isoforms expressed in oocyte pairs, showing the following decreasing order: Cx50 > Cx46 > Cx45 > Cx26 > Cx37 > Cx43 > Cx40 > Cx32 [104].

The sensitivity of Cx43 to intracellular acidification seems to depend on the CT region of the protein, as its truncation abolishes pH regulation [101]. Furthermore, pH sensitivity was restored when pH-insensitive channels, lacking the CT domain, were coexpressed, in Xenopus oocyte pairs, with mRNA coding for the CT of the protein [102]. Dependence on the CT domain was later confirmed for other connexin isoforms, such as connexins 37, 40 and 50 [104], but not for Cx26 [105]. Interestingly, these studies also demonstrated that the CT domain of Cx40 is able to regulate channels formed by truncated Cx43 and vice versa [104]. All these findings support the notion that pH/chemical gating can be dependent on a “ball-and-chain” mechanism, similar to that previously described for fast Vj gating [97,106]. Deletion mutagenesis has allowed mapping proline-rich repeats within the CT domain of Cx43 as important for pH gating [103]. Furthermore, a histidine residue at the interface between the second transmembrane domain and the cytoplasmic loop has been implicated as the receptor for the CT domain [107], although other possibilities have also been discussed [4]. In fact, some controversy exists as to whether pH gating can be mediated by direct protonation of that specific histidine residue within the cytoplasmic loop [85,108], or by indirect mechanisms through protonation of endogenous aminosulfonates, such as taurine in intact cells [16].

Interestingly, intramolecular interactions between the CT domain of Cx43 and its intracellular loop have also been described to gate unopposed Cx43 hemichannels. However, in contrast to gap junction channels, this interaction seems to facilitate opening of free hemichannels [8]. Such differences in pH regulation between gap junctional channels and free hemichannels are in agreement with previous studies showing that deletion of the Cx43 carboxiterminus allows formation of functional gap junction channels, but not that of functional hemichannels [8,109].

Gating by intracellular acidosis of Cx43, Cx40 and Cx45 gap junctional channels has been suggested to play a key role in cell-to-cell uncoupling occurring during ischemia, a process in which the CT domain of the molecule plays a critical role [110]. Thus, similar to Vj and Vm gating, pH gating might constitute one of the mechanisms responsible for electrically isolating ischemic regions during myocardial infarction.

#### 2.3.3. Calcium- and Calmodulin-Dependent Gating

It is widely admitted that increasing the intracellular calcium concentrations uncouples gap junctions in most tissues [13,111,112,113]. Early studies demonstrated that intracellular calcium injections induced electrical uncoupling [114,115,116]. The sensitivity of gap junctional channels to intracellular Ca^2+^ concentrations depends not only on the connexin isoform but also on the cell type [111]. However, it is still unclear whether the effects of Ca^2+^ are direct or due to the action of intracellular mediators. In this sense, several pieces of evidence indicate that Ca^2+^ probably induces gap junction closure by activation of calmodulin, which may act directly as a gating particle [111,117,118,119]. Interestingly, calmodulin has been shown to colocalize and to interact with several connexin isoforms, including cardiac Cx43 and Cx45 [85,120]. The role of calmodulin in calcium-dependent gating has been strengthened by the demonstration that its inhibition prevents uncoupling in a number of cell types [93,111,119]. In the case of Cx43, initial studies suggested that calmodulin binds the cytoplasmic loop, in a region overlapping the binding receptor site (L2 region) for the CT domain [121], thus inducing channel closure through the particle-receptor mechanism. However, recent studies conducted in neuroblastoma-2a cells and in neonatal mouse ventricular myocytes expressing the Cx43-M257 (Cx43K258stop) mutant protein, a CT-truncated version of Cx43 lacking pH sensitivity, demonstrated that these cells are still gated by calcium/calmodulin [93]. These findings thus support that calcium- and calmodulin-dependent gating of Cx43 is independent of the distal CT domain [93]. Furthermore, although some works suggested the existence of synergies between calcium and protons [122,123], this study also demonstrated that both phenomena are independent [93].

A distinct characteristic of unopposed connexin hemichannels, in comparison with gap junctional intercellular channels, is that they are regulated by variations in concentrations of both intracellular and extracellular calcium [124]. In a seminal paper, DeVries and Schwartz demonstrated that a reduction in extracellular cations in general, and specifically in calcium, markedly activates connexin hemichannels, whereas exposure to calcium reduces their open probability [125]. This finding was later confirmed in most connexin isoforms, including Cx43 [124,126,127,128,129]. In fact, homomeric Cx43 hemichannels rapidly and reversibly increase their internal pore diameter from 1.8 to 2.5 nm upon extracellular calcium removal, as demonstrated by high-resolution atomic force microscopy [130]. The exact mechanisms by which extracellular calcium, including physiological concentrations, blocks connexin hemichannels are not well understood. However, it is unlikely that it takes place just due to conformational changes in the molecule and rather involves direct cation binding to the mouth of the hemichannel pore [124]. On the other hand, intracellular calcium has been demonstrated to induce a bell-shaped-dependent effect on hemichannel opening [131,132]. An increase in intracellular calcium triggers hemichannel opening by multiple signaling steps, including calmodulin, CaMKII and reactive oxygen species (ROS), with maximal conductance reached at concentrations around 500 nM [131,132]. However, higher intracellular calcium concentrations induce closure of connexin hemichannels [131,132].

As seen for voltage and pH, calcium gating of gap junctional intercellular channels might also constitute a protective mechanism in the heart, thus electrically isolating injured tissue during ischemia. Furthermore, it may also prevent the spreading of injury between neighboring cells, attenuating leakage of metabolites [85]. This phenomenon, initially described in cardiac tissue and termed as “healing over” by Engelmann in 1877 [133], caused him to notice the existence of differences between cardiac and skeletal muscle cells. In his words, “cardiomyocytes live together but die alone” [133]. However, as described before, the counterpart can be an increased incidence of reentrant ventricular arrhythmias [134]. Gating by intracellular calcium, similar to pH, has been demonstrated to play a key role in cell-to-cell uncoupling of Cx43 channels during ischemia [110].

### 2.4. Regulation by Post-Translational Modifications

Post-translational modifications regulate multiple aspects of connexins’ life cycle, including synthesis, trafficking, assembly into gap junctions, degradation and gating, and have been previously reviewed elsewhere [8,26,42,135,136]. The best-known and, probably, the most important post-translational modification is phosphorylation, but others include acetylation, S-nitrosylation, ubiquination and SUMOylation. Some of these changes, including phosphorylation, may modify the net charge of the protein, which, in turn, may also alter the voltage or pH sensitivity of the channels. Here, we are going to summarize the most important knowledge regarding regulation of the main cardiac connexins (i.e., Cx43, Cx40 and Cx45).

#### 2.4.1. Phosphorylation

**Figure 4:**
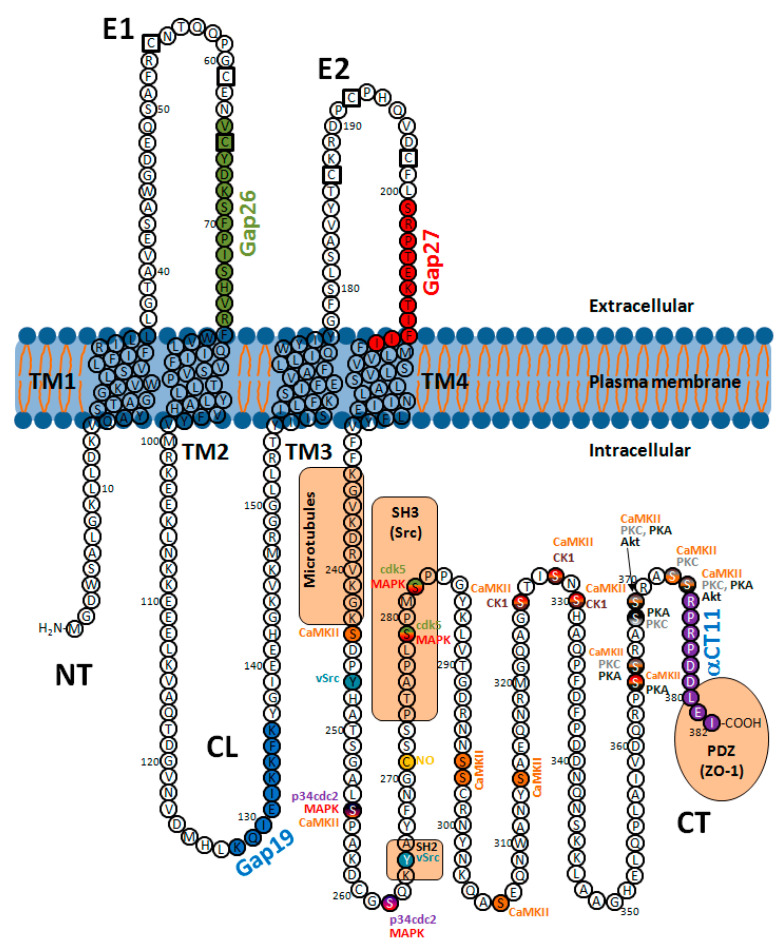
Human Cx43 amino acid sequence, showing location of proposed regulatory residues. Conserved cysteines in the extracellular loops (E1 and E2), important for the docking of opposing hemichannels, are shown in black squares. Interacting sequences with microtubules, zonula occludens-1 and Scr are shown inside pink boxes. Location of inhibitory mimetic peptides Gap26, Gap27 and Gap19 are shown in green, red and blue, respectively. In addition, the last 9 residues of the 25-amino acid Cx43-regulatory peptide αCT1 are shown in purple (this 9-amino acid peptide is known as αCT11). CL, NT and CT indicate cytoplasmic loop, and amino- and carboxyterminal domains, respectively. Modified from [11,138,143,144].

Most connexin isoforms, including Cx26, Cx31, Cx32, Cx36, Cx37, Cx40, Cx45, Cx46, Cx50 and Cx56, are phosphoproteins, all of them having multiple phosphorylation sites [136,137,138,139]. This is also the case for Cx43, the best studied connexin isoform. At least 21 different phosphorylation consensus sites have been described for this connexin isoform (Figure 4) [42,140,141], although others may also exist [141]. In general, most phosphorylation events occur primarily within the CT domain of the molecule [8,138,141,142]. Additionally, a putative phosphorylation site at the NT domain of Cx43 (Ser5) has also been identified by mass spectrometry [141].

**Figure 5:**
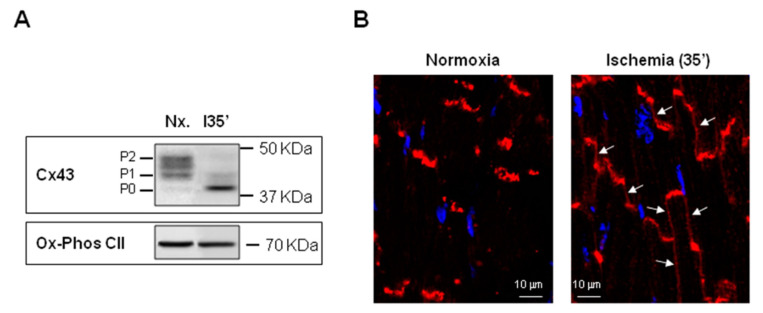
(**A**) Western blot showing expression of Cx43 in isolated mice hearts submitted to normoxic perfusion or to 35 min of global ischemia. Mitochondrial respiratory complex II was used as loading control. (**B**) Distribution of Cx43 in cardiac slices obtained from isolated mice hearts submitted to the same protocols. Arrows indicate lateralized gap junctions appearing under ischemic conditions.

From the 21 phosphorylation sites identified at the CT domain of Cx43 (Figure 4), 18 correspond to serines, 1 to threonine and 2 to tyrosines, with some of these residues being targeted by multiple protein kinases [42,140,141]. Cx43 phosphorylation events can occur shortly after synthesis [145] and regulate not only the channel molecular structure and gating (modifying the open probability and unitary conductance) but also connexin synthesis, intracellular trafficking, assembly into gap junctional plaques and degradation, thus conditioning the net effect on GJIC [7,8,11,26,42,87,135,136,138,146]. Cx43 migrates as multiple electrophoretic species when analyzed by SDS-PAGE, with most cell types displaying at least three different bands, often labeled as P0, P1 and P2 (Figure 5) [140,147]. The faster-migrating Cx43 species, P0, appears as a band at a molecular weight of about 42 kDa and is considered as a non-phosphorylated state, as it is not metabolically labeled with [^32^P]orthophosphate in intact cells [147]. On the other hand, the P1 and P2 species migrate between 44 and 46 kDa and represent different phosphorylation states, as demonstrated by their positive labeling with [^32^P]orthophosphate and by the fact that they are converted to the P0 state after alkaline phosphatase treatment [147]. Pulse-chase experiments seem to indicate that newly synthesized Cx43 migrates at P0 and then sequentially matures to P1 and P2 [147]. Transfer of Cx43 hemichannels to the plasma membrane is thought to involve phosphorylation on Ser364/365, an event inducing a conformational change visible as P1 by SDS-PAGE [28]. Later on, casein kinase 1 (CK1) phosphorylation on Ser325/328/330 is thought to facilitate the transition from the membrane into the gap junctions, at the intercalated disk, thus creating the P2 band [148,149]. However, these shifts in electrophoretic mobility, of about 2–4 kDa, cannot simply be due to the addition of a single phosphate, which has a comparative small mass of only 80 Da [140]. Finally, migration as multiple bands is also apparent in other cardiac connexins, such as Cx45 and Cx40. Cx45 appears as two distinct species migrating at 46 and 48 kDa, with the slower form shifting to the 46 kDa state after alkaline phosphatase treatment [150]. Cx40 is normally detected as a band at 40 kDa, but treatment with cAMP might give rise to a second band at 42 kDa [151].

##### Cx43 Channels

Phosphorylation of Cx43 by PKC occurs at several amino acids, including Ser365, Ser368, Ser369, Ser372 and Ser373 (Figure 4) [11,87,138,152,153,154]. The main consequences of an enhanced PKC-dependent phosphorylation of Cx43 are a diminished hemichannel assembly, a decrease in gap junction intercellular communication and a reduction in the half-life of the protein [135,138,155]. Phosphorylation of Ser368 by PKC seems to have a prominent role in these effects [138,153]. Thus, whereas an enhanced PKC-dependent phosphorylation of Ser368 after treatment with lysophosphatidylcholine or cholesterol in H9c2 cells was shown to be associated with a reduction in gap junctional communication [154,156], an attenuated phosphorylation of this residue, either by direct PKC inhibition or after treatment with simvastatin, prevented such effect [156,157]. The reduced coupling secondary to Ser368 phosphorylation has been shown to be associated with ubiquitination of the protein and gap junction disassembly [156], whereas preventing Ser368 phosphorylation increased Cx43 localization at the cell membrane [157]. Finally, in addition to changes in hemichannel assembly and redistribution of the protein, PKC phosphorylation also regulates Cx43 gating. Site-directed point mutation experiments demonstrated that PKC-dependent Ser368 phosphorylation induces a shift in the unitary conductance of Cx43 channels, with a large increase in the low-conductance state (about 50 pS) and a concomitant loss of the 100 pS channel events [158]. Nonetheless, this does not necessarily translate to a decrease in macroscopic junctional conductance. In fact, experiments in transfected cells, using patch clamp techniques, demonstrated an increase in total conductance of about 45%, despite a reduction in unitary conductance and permeability [89]. This might indicate that a large number of channels were incorporated and activated or that the open probability of the already active channels increased during kinase activation, with the last possibility having been suggested to be unlikely [87]. Finally, in addition to its well-known effects on gap junction communication, PKC-mediated phosphorylation also directly regulates the function of unopposed hemichannels. Thus, PKC inhibition has been demonstrated to increase Cx43 hemichannel activity, enhancing permeant uptake [153,159,160,161]. In fact, the PKC-activating phorbol ester 12-myristate 13-acetate (PMA) has been shown to markedly attenuate Cx43 hemichannel currents, an action antagonized by general PKC inhibitors and by selective PKCε inhibitors [161]. The main regulatory site involved in PKC modulation of Cx43 hemichannels is also Ser368 [160,161].

Cx43 appears to be a relatively poor substrate for purified cAMP-dependent protein kinase A (PKA), as compared with protein kinase C (PKC) [162]. Despite this, it is widely admitted that Cx43 is phosphorylated by PKA at Ser364 (Figure 4), increasing gap junction assembly [163]. This effect seems to be mediated by an enhanced trafficking of connexons from intracellular stores to the plasma membrane, with no increase in total levels of Cx43 [164]. The final result is a rapid increase in gap junctional communication [165,166]. Furthermore, phosphorylation at Ser364 by PKA appears to be necessary for subsequent multiple phosphorylations by PKC [162]. However, Ser364 is not the only target of PKA. Ser365, a residue mainly targeted by PKC, might also be phosphorylated by PKA [167]. Moreover, treatment with follicle-stimulating hormone (FSH), which elevates intracellular cAMP concentrations, has been shown to enhance Cx43 phosphorylation at Ser365, Ser368, Ser369 and Ser373, together with protein levels, in rat primary granulose cells [168]. These residues fit well with putative PKA phosphorylation sites [168], but, on the other hand, cAMP may act through PKA-independent mechanisms in this cell type [169]. Further studies are needed to confirm or discard a role for PKA-mediated phosphorylation of these residues in Cx43.

Studies in transgenic mice have allowed demonstrating that Cx43 is a target of Akt (protein kinase B, PKB) [170]. Two potential Akt phosphorylation sites, Ser369 and Ser373, have been described on the Cx43 CT domain (Figure 4) [171,172]. Akt phosphorylation of Cx43 at Ser373 stabilizes and increases the size of gap junction plaques [65]. This effect seems to be mediated by preventing the interaction between Cx43 and zonula occludens-1 [173]. Thus, phosphorylation on S373 has been proposed to act as a molecular switch to rapidly increase gap junctional communication under some situations, including ischemia [173]. Furthermore, in addition to regulation of gap junction channels, Akt is also able to regulate free, unopposed hemichannels [172]. Direct Akt phosphorylation of Cx43 at Ser373 has been shown to induce opening of Cx43 hemichannels, enhancing interaction with integrin α5 in osteocytic MLO-Y4 cells, a process critical in bone formation [172].

Gap junctional communication through Cx43 channels is depressed by drugs that enhance intracellular levels of cyclic guanosine 3′,5′-monophosphate (cGMP), such as 8-bromo-cGMP or carbachol [166,174]. Moreover, cGMP treatment has been shown to shift, in whole cell experiments carried out in pairs of rat neonatal cardiomyocytes, single-channel conductance from the higher-conductance state to the lower substate, a change that was prevented by intracellular addition of phosphatases [174]. The last finding might be indicative of a cGMP-mediated stimulation of protein kinase G (PKG). Indeed, the intracellular CT domain of Cx43 contains putative consensus motifs for PKG-mediated phosphorylation [11]. The decrease in macroscopic and single-channel conductance observed in SKHep1 cells and rat cardiomyocytes treated with 8-bromoguanosine 3′:5′-cyclic monophosphate (8Br-cGMP) seems to be due to cGMP-dependent phosphorylation of Cx43 on Ser257 [175,176]. However, whereas Ser257 phosphorylation by PKG seems to regulate electrical coupling in the rat, this might not be the case in humans, as human Cx43 contains an alanine instead of a serine in that residue [87].

Up to 15 different serines within the CT of Cx43 have been identified as putative phosphorylation sites for CaMKII [11,138,177]. They include residues 244, 255, 257 (not in humans), 296, 297, 306, 314, 325, 328, 330, 364, 365, 369, 372 and 373, as identified by mass spectrometry (Figure 4) [177]. CaMKII regulation of Cx43 can be especially important in cardiac pathophysiology, and it may be, at least in part, responsible for some of the uncoupling effects ascribed to calcium and calmodulin [138]. In fact, heart failure and myocardial infarction have been associated with enhanced expression of CaMKII [177], and enhanced colocalization of CaMKII and Cx43 has been demonstrated at the infarct border zone in dogs [178]. On the other hand, however, Ser306 dephosphorylation occurs during myocardial ischemia in rat hearts and may contribute to reduced gap junctional coupling under this condition [179]. Indeed, a serine-to-threonine mutation at Ser306, a modification that renders this residue unable to be phosphorylated, induced a reduction in macroscopic conductance in HeLa cell pairs [9]. This effect was not caused by changes in Cx43 abundance or distribution but was associated with a marked reduction in the main unitary conductance state and a concomitant increase in the lower-conductance substates [9].

Cx43 is also a target for casein kinase 1 (CK1). CK1 has been shown to interact with and to phosphorylate Cx43 at Ser325, Ser328 and Ser330 in normal rat kidney (NRK) cells (Figure 4) [148]. Analysis of Cx43 content after CK1 inhibition demonstrated a slight increase in total Cx43, whereas gap junctional Cx43 was decreased and non-junctional plasma membrane Cx43 increased [148]. These results suggest that Cx43 phosphorylation by CK1 may regulate gap junction assembly.

Cx43 can be phosphorylated by the mitogen-activated protein kinase (MAPK) pathway at Ser255, Ser262, Ser279 and Ser282 (Figure 4) [180,181,182]. This family of kinases includes a variety of proteins, such as extracellular signal-regulated protein kinases 1 and 2 (ERK1/2), p38 MAPK, c-Jun N-terminal kinase (JNK) and many others [183]. Cx43 phosphorylation at these four residues after exposure to vascular endothelial growth factor (VEGF) has been shown to be dependent on ERK1/2 activation in porcine pulmonary artery endothelial cells, an effect associated with loss of Cx43 at gap junctions and a concomitant increase in its cytoplasmic expression, resulting in inhibition of gap junctional communication [182]. Similarly, activation of the MAP kinase pathway by EGF or lysophosphatidic acid was demonstrated to disrupt gap junctional communication through phosphorylation on Ser255, Ser279 and Ser282 in different cell lines [180]. Furthermore, cells expressing a mutant Cx43 that cannot be phosphorylated on Ser279 and Ser282 show restoration of gap junction assembly, through prevention of clathrin-mediated Cx43 endocytosis [184]. On the other hand, inhibition of gap junctional communication after EGF treatment was shown to be independent of a reduction in unitary channel conductance [185]. Thus, and although effects in the open channel probability or in perm selectivity cannot be discarded, these results seem to indicate a prominent role of MAPK-mediated phosphorylation in Cx43 internalization and gap junction assembly. In addition, MAPK may also influence Cx43 unopposed hemichannels, reducing its permeability to fluorescent probes, as demonstrated in liposomes containing Cx43 [186].

Other kinases regulating Cx43 function and turnover through serine phosphorylation include cyclin-dependent kinase 5 (Cdk5) and cyclin-dependent kinase p34cdc2. The gradual decrease in Cx43 content occurring in the brain cortex during embryonic development was demonstrated to correlate with upregulation of Cdk5 activity. In fact, Cdk5 has been shown to phosphorylate Cx43 at Ser279 and Ser282 (Figure 4), preventing membrane targeting of the protein and promoting proteasome-dependent degradation [187]. However, its role in the myocardium has not been investigated. In addition, the reduction in gap junctional communication seen in mitotic cells is associated with increased phosphorylation of Cx43 at Ser255 and Ser262 due to enhanced p34cdc2 activity (Figure 4) [188]. This phosphorylation event has been related to gap junction internalization and the appearance of an additional electrophoretic Cx43 band (P3) migrating slower than the P2 species [188,189]. Again, the role of the p34cdc2-dependent phosphorylation of Cx43 in the myocardium is unknown.

In addition to regulation by serine/threonine protein kinases, Cx43 gap junction channels are modulated by several tyrosine protein kinases, including v-Src [87,138,190]. Members of the Src family of protein kinases are products of proto-oncogenes that play key physiological roles in cell morphology, motility, division and survival [191]. The viral Src (v-Src) is encoded by an oncogen found in the Rous sarcoma retrovirus, which affects chickens, whereas the cellular homologue (c-Scr), the first discovered proto-oncogene, is encoded by the SCR gene [191]. All of them are non-receptor protein tyrosine kinases [191] and have been long reported to downregulate gap junction communication [192,193]. Studies using in vitro kinase reactions demonstrated that purified v-Scr is able to phosphorylate Cx43 [194], and both proteins have been shown to coprecipitate in v-Scr-transformed cells [195], and to interact both in vivo and in vitro [195,196]. The available evidence seems to indicate that the SH3 and SH2 domains of v-Scr bind to proline-rich motifs (Pro274–Pro282) and phosphorylate tyrosine residues within the CT domain of Cx43 [195,196]. Indeed, phosphorylation of Cx43 by v-Scr kinase occurs at Tyr247 and Tyr265 (Figure 4) [185,197]. It has been proposed that the SH3 domain of Scr would initially bind to the proline-rich region of Cx43, bringing the Scr kinase domain close to Tyr265, allowing its phosphorylation [197]. This event, in turn, would facilitate binding of the SH2 domain of Scr, stabilizing the interaction with Cx43, allowing phosphorylation of Tyr247, and triggering the closure of gap junction channels [197]. The net results of these phosphorylation events are a marked reduction in gap junction permeability to fluorescent dyes [145,198], a modification in chemical selectivity [185] and a decrease in macroscopic conductance [185]. However, no major changes in unitary channel conductance have been observed after exposure to v-Scr [185]. Moreover, those effects have been proposed to also be independent of changes in the number of available channels [87], as comparable levels of Cx43 were observed in cells expressing or not expressing v-Scr [197]. Taken together, these data suggest, as the only plausible explanation for the reduction in macroscopic conductance seen after exposure to v-Scr, that the kinase induces a decrease in the open probability of the channels [87].

##### Cx40 Channels

Less known are the effects of phosphorylation events in the regulation of other cardiac connexins. In this sense, PKA has been shown to modulate Cx40 [151,199]. Administration of cAMP was described to increase macroscopic gap junction conductance and permeability in a Cx40-transfected, communication-deficient human hepatoma cell line (SKHep1 cells) [151]. This effect seems to be mediated by a shift from the single-conductance state of 80 pS to the higher-conductance state of 120 pS [151], suggesting that PKA would modulate Cx40 gating.

##### Cx45 Channels

The PKC-activating phorbol ester PMA has been shown to increase macroscopic junctional conductance in HeLa cells transfected with mouse Cx45 by about 50%, whereas cAMP was shown to induce a modest reduction in gap junctional conductance of about 20% of the initial value [150]. However, these effects were not associated with changes in the unitary conductance of Cx45 channels, which showed, in both circumstances, two conductance peaks of about 20 and 40 pS of similar magnitude, nor with differences in expression of the protein [150]. A possible explanation was that both maneuvers modified the open probability of the channels.

In addition, Cx45 can also be a target for CaMKII, which would phosphorylate up to eight different serines or threonines (Ser326, Thr337, Ser 381, Ser382, Ser384, Ser385, Ser387 and Ser393) [200]. Five of these residues (Ser326, Ser382, Ser384, Ser387 and Ser393) were shown to also be phosphorylated by CK1 [200]. Finally, Cx45 is also regulated by tyrosine kinases. Thus, it has been demonstrated that HeLa cells expressing exogenous mouse Cx45, when treated with pervanadate, a tyrosine phosphatase inhibitor, depicted a reduction in cell coupling of about 40% [150]. This effect was associated with an increase in Cx45 phosphorylation, was independent of changes in unitary channel conductance and was probably secondary to modulation of the open channel probability [150]. However, the exact nature of the tyrosine kinase responsible for these effects is unknown [87].

#### 2.4.2. Redox Regulation: S-Nitrosylation and Carbonylation

Changes in redox potential are common regulatory mechanisms of membrane ion channels, including those formed by connexins [138,201,202]. Three oxidant gaseous transmitters, nitric oxide (NO), carbon monoxide (CO) and hydrogen sulfide (H_2_S), have been described to modulate the function of either gap junction channels or undocked hemichannels.

NO may influence protein function in two different ways: through direct S-nitrosylation of the molecule, or, indirectly, through activation of guanylyl cyclases, increasing cGMP levels and activating PKG [138]. Remarkably, protein S-nitrosylation does not occur in all cysteine residues available, as it is highly dependent on the cysteine oxidation state and on the surrounding amino acids [26]. In the case of connexins, cysteine residues located at the extracellular loops have been demonstrated not to be S-nitrosylation targets [203].

S-nitrosylation of Cx43 has been suggested to be involved in the regulation of the myoendothelial junction in resistance vessels [204]. In fact, it was shown that active endothelial NO synthase is enriched at the myoendothelial junction in these arteries in mice, resulting in constitutive S-nitrosylation of Cx43 on cysteine 271 (Figure 4), located at the intracellular CT domain [204]. The final result of Cx43 S-nitrosylation at gap junctions seems to be an enhanced junctional permeability, as suggested by the finding that pharmacological inhibition or genetic depletion of the denitrosylase increases gap junction communication at the myoendothelial junction [204]. Furthermore, S-nitrosylation of gap junctional channels has been suggested to explain, at least in part, the reduction in the severity of ischemia-induced arrhythmias, and of ischemia-induced changes in myocardial electrical resistivity, occurring after treatment with sodium nitrite in dogs submitted to transient coronary occlusion [205]. In addition, S-nitrosylation has been suggested to also regulate the permeability of undocked Cx43 hemichannels, probably playing a role in cell responses to hypoxia, oxygen deprivation or long periods of ischemia [137]. In this sense, it is widely admitted that metabolic inhibition induces the opening of free hemichannels in cortical astrocytes and cardiomyocytes [128,129,206]. Moreover, metabolic inhibition may increase the number of free hemichannels at the cell membrane in cortical astrocytes, an effect associated with increased dephosphorylation and S-nitrosylation, and ultimately leading to an increase in cell permeability [207]. Interestingly, treatment with reducing agents did not affect Cx43 phosphorylation but reduced both dye uptake and Cx43 S-nitrosylation [207]. Moreover, exogenous NO donors enhanced the cell permeability and S-nitrosylation of surface Cx43 but did not affect its abundance or phosphorylation [207]. Taken together, these findings may suggest a role for S-nitrosylation in the regulation of the Cx43 hemichannel open probability and permeability [136,207]. However, differences may exist depending on the metabolic status of the cell, as reducing agents show opposite effects (i.e., increased hemichannel opening) in normoxic cells, possibly by acting on cytoplasmic cysteine residues within the CT domain [208]. On the other hand, S-nitrosylation of mitochondrial Cx43 has been proposed to contribute to the protective effect of ischemic preconditioning, a cardioprotective maneuver consisting of brief episodes of ischemia/reperfusion applied before a longer, and potentially lethal, index ischemia [209].

CO donors have been shown to inhibit Cx43 and Cx46 hemichannel currents and dye uptake in HeLa and MCF-7 cells [210]. In the case of Cx46, this effect was attenuated by reducing agents acting extracellularly. In fact, Cx46 hemichannel inhibition was shown to be dependent on direct carbonylation of the protein, an action in which cysteines located at the extracellular loops are important [210]. Taken together, these findings suggest a direct regulatory effect of CO on Cx46, although this has not been proven yet for Cx43.

Cx43 can also be reversibly oxidized at cysteine 260, located within the CT domain of the molecule [211]. This redox modification, which has been suggested to be dependent on Toll-like receptor 2 (TLR2) activation, might be important under pathological situations, such as during myocardial ischemia/reperfusion. Administration of H_2_O_2_ or TLR2 agonists to cultured HL1 cardiomyocytes has been shown to accelerate action potential propagation, whereas TLR2^−/−^ mice, which had reduced susceptibility to ischemia/reperfusion injury [212], depicted a reduced mitochondrial ROS production and attenuated Cx43 oxidation at Cys260, as compared with wild-type animals [211]. Furthermore, oxidative stress, induced by several treatments, including H_2_O_2_, has been demonstrated to open undocked hemichannels in Marshall cells expressing only Cx43, compromising cell survival [213].

#### 2.4.3. Acetylation

Cx43 has been shown to become N-lysine acetylated in hearts from the mdx mouse model of Duchenne dystrophinopathy, and to colocalize with both the histone acetylase PCAF and class I and IIa histone deacetylases [214]. Hyperacetylation of Cx43 was associated with dissociation from gap junctions and redistribution along the long axis of ventricular cardiomyocytes, including the cytoplasm and the nucleus [214]. Prediction analysis demonstrated that at least three putative lysine N^ε^ acetylation sites exist at the molecule, one at the N-terminus (Lys9) and two at the CT domain (Lys234 and Lys264) [214]. Furthermore, gap junction remodeling and the consequent reduction in cell-to-cell communication following cardiac pacing have been associated with increased Cx43 acetylation [215].

#### 2.4.4. SUMOylation

Cx43 has been shown to be covalently modified by members of the small ubiquitin-like modifier (SUMO) family of proteins [216]. The available evidence indicates that such post-translational modification occurs at lysine residues 144 and 237, located at the intracellular loop and the CT domain, respectively [216]. Overexpression of all three SUMO isoforms (SUMO-1/-2/-3) in HeLa cells has been shown to increase total Cx43 expression, to promote gap junction formation and to enhance dye transfer between neighboring cells [216]. On the other hand, mutations of Lys144 or Lys237 lead to a reduced ability to form gap junctions [216]. Although the exact mechanisms by which SUMOylation regulates connexin expression are not known, these findings suggest that this post-translational modification might be involved in stabilization of gap junctions at the plasma membrane [216].

#### 2.4.5. Ubiquitination

The human Cx43 has 10 lysine residues at the CT domain, all of which may constitute potential ubiquitination sites [8]. Additionally, other cytosolic lysines might also be susceptible to this modification [217]. Initial studies using sequential immunoprecipitation analysis with anti-Cx43 and anti-ubiquitin antibodies suggested that Cx43 was polyubiquitinated in E36 Chinese hamster ovary cells [51]. Later on, others demonstrated, instead, and using different antibodies able to differentially recognize polyubiquitinated and monoubiquitinated proteins, that Cx43 is multimonoubiquitinated in NRK cells and in TPA-treated rat liver epithelial IAR20 cells [56,59]. Nonetheless, ubiquitination occurs through the action of several E3 ubiquitin ligases, including Trim21 [58], WWP1 [218], SMURF2 [219] and NEDD4 [59,220]. All of them have been associated with gap junction plaques, and they may play a key role in gap junction internalization and degradation [50]. Supporting the importance of ubiquitination in connexin degradation, expression of a chimeric Cx43 with a fused ubiquitin molecule at the CT domain, mimicking Cx43 ubiquitination, forms smaller gap junctions and has a higher turnover rate than wild-type Cx43 [221].

### 2.5. The Connexin Interactome or Connexome

Connexins, and specifically Cx43, have been shown to interact, mainly through their CT domain, with a wide variety of proteins [8,13,35,144,222,223,224,225]. A list of Cx43-interacting proteins validated by classical biochemical and microscopy techniques can be found in [144,225,226]. Some of these interactions are related to the regulation of the connexin function and include calmodulin, protein kinases and E3 ubiquitin ligases, as discussed in previous sections. However, connexins can also interact with components of the cytoskeleton as well as with various scaffolding proteins, which facilitate such interaction, but also with tight junction-associated proteins, with anchoring junction-associated proteins and with membrane channels and receptors [8,13,35,144,222,223,224,225]. Here, we are going to summarize some of the best-known interactions. For comprehensive discussions on the connexin interactome, the reader is referred to some excellent reviews in the field [8,13,35,144,222,223,224,225].

#### 2.5.1. Interactions of Sarcolemmal Connexins with Cytoskeletal Proteins

Previous studies have demonstrated that the Cx43 CT domain interacts directly with microtubules [227], in a process that is inhibited by phosphorylation at Tyr247, a site known to be targeted by the tyrosine kinase Src [8,228]. A 35-amino acid juxtamembrane region within the Cx43 CT domain contains a presumptive tubulin-binding motif (Figure 4) and is probably responsible for this interaction [227]. Cx43 binds equally well to both α- and β-tubulin [229]. Interaction between Cx43-formed gap junctions and the distal end of microtubules might be important to modulate cell polarity and directional cell migration. In this regard, cardiac defects in Cx43 knock-out mice might be due to disruption of Cx43–tubulin interactions and alterations in cell polarity during embryonic development [230]. As we have discussed before, microtubules mediate targeted delivery of Cx43-containing vesicles to the plasma membrane [27].

In contrast to its direct binding to microtubules, Cx43 interaction with the actin-based cytoskeleton is thought to be indirect and mediated through adaptor proteins, including zonula occludens-1 (ZO-1) and debrin (developmentally regulated brain protein) [8,225]. Adaptor proteins usually bind to the CT domain of different transmembrane proteins, connecting them to the actin cytoskeleton, either directly, or through recruitment of other proteins (e.g., α-catenin or afadin), thus creating a multiprotein complex [225]. Zonula occludens-1 fulfills these characteristics and has been shown to interact with all connexin isoforms analyzed until date, including Cx43, Cx40 and Cx45 [225]. Cx43 interacts, through its CT domain, with the second PDZ domain of zonula occludens-1 (Figure 4) [231,232], and this interaction occurs predominantly at the gap junction periphery, within the perinexus [35,36,233]. It has been proposed that zonula occludens-1 controls the rate of Cx43 hemichannel accretion at the gap junction periphery, thus participating in regulation of the gap junction size and distribution [234]. Indeed, blockade of the Cx43 and zonula occludens-1 interaction led to a reduction in their association specifically at the gap junction periphery, together with an increase in the plaque size, due to accumulation of gap junctional channels from non-junctional pools [234]. As mentioned above, Akt phosphorylation of Cx43 at Ser373 prevents this interaction, thus increasing the gap junction size [65,173]. However, and despite its involvement in constraining gap junction growth, zonula occludens-1 is dispensable for assembly of connexons into gap junctions, as CT-truncated versions of Cx43, lacking the zonula occludens-1 binding domain, can still form functional channels [235,236]. Similarly to zonula occludens-1, debrin also interacts with the CT domain of Cx43 [237,238]. Indeed, debrin depletion with siRNA results in impaired cell-to-cell coupling, internalization of gap junctions and targeting of Cx43 to degradation [237]. These data would suggest that debrin plays a role in maintaining gap junctions in a functional state. The debrin binding sites do not overlap with those of zonula occludens-1 and tubulin [238]. Accordingly, simultaneous binding of all these proteins would create a supramolecular complex that might be important in regulation of cytoskeleton rearrangements [238].

#### 2.5.2. Interactions of Sarcolemmal Connexins with Other Junctional Proteins

Despite the fact that gap junctions and tight junctions may represent different specialized plasma membrane microdomains, they share several adaptor proteins. Tight junctions constitute a semipermeable barrier that regulates the flux of ions, solutes and cells across paracellular spaces [239]. They are formed by the transmembrane proteins claudins and occludins, but also by several PDZ domain-containing proteins, including zonula occludens, and by additional proteins lacking the PDZ domain [239]. In this regard, Cx43 and Cx40 have been shown to coprecipitate and colocalize with occludin, claudin-5 and zonula occludens-1 in porcine blood–brain barrier endothelial cells [240].

Integrins, cadherins and cell adhesion molecules, such as selectins, are transmembrane proteins that are part of anchoring junctions (desmosomes and adherens junctions). They, together with gap junctions, are included within the intercalated discs of cardiomyocytes and mediate cell-to-cell and cell-to-matrix interaction and communication. Cadherins are linked to the actin cytoskeleton through catenins, a group of multifunctional proteins including α-catenin, β-catenin, plakoglobin and p120 catenin [241]. Cx43 has been suggested to co-assemble in a multiprotein complex with N-cadherin, p120 catenin and several other N-cadherin-associated proteins in NIH3T3 cells [242]. A similar complex including α-catenin, β-catenin, zonula occludens-1 and Cx43 has been found in rat neonatal cardiomyocytes [243]. This complex might be required for Cx43 transport to the plasma membrane during gap junction assembly [243]. These interactions might be important, thus, for proper gap junction and adherens junction formation [242]. Other components of anchoring junctions that interact with Cx43 at the intercalated discs include ankyrin-G and plakophilin-2 [244,245]. Mutations in the desmosomal protein plakophilin-2 have been associated with arrhythmogenic right ventricular cardiomyopathy [246]. On the other hand, interaction of the Cx43 CT domain with intergrin α5β1 has been shown to be required for proper opening of unopposed hemichannels under mechanical stimulation in osteocytes [247].

Vinculin, a cytoplasmic actin-binding protein that anchors the actin cytoskeleton to the plasma membrane, and which is present in cell–matrix adhesions and in adherens junctions, is also essential for maintaining gap junctional intercellular communication in cardiomyocytes [248]. Vinculin, Cx43 and zonula occludens-1 were found to colocalize at intercalated discs in mouse neonatal cardiomyocytes [248]. Excision of the vinculin gene led to a reduction in Cx43 and zonula occludens-1 expression and altered gap junctional intercellular communication [248].

#### 2.5.3. Interactions of Sarcolemmal Connexins with Receptors and Ion Channels

Connexins, and specifically Cx43, have been found to interact with a number of membrane channels and receptors. These include muscarinic acetylcholine receptors, purinergic receptors and receptor protein tyrosine phosphatases [225]. Especially relevant to cardiac electrophysiology is the interaction with Na_v_1.5 sodium channels, which provide the current necessary for generation of the action potential in most cardiac cells [249,250]. It has been proposed that two distinct pools of Na_v_1.5 channels exist in the heart, one located at the intercalated disc, and the second one being associated with the costameres [251]. The importance of this interaction is demonstrated by the fact that sodium currents are larger in the intercalated disc area and in cells that remain paired [252]. In addition, gap junctional intercellular coupling is affected by loss of plakophilin-2 [253], a situation that also alters sodium channel function [254]. The relationship between Cx43 and Na_v_1.5 channels at the perinexus was further supported by the finding that a reduced Cx43 expression in conditional Cx43^Cre-ER(T)/fl^ knock-out mice was associated with diminished Na_v_1.5 expression and attenuated sodium currents [255]. Furthermore, immunofluorescence analysis using the Duolink proximity ligation assay confirmed the association between Cx43 and Na_v_1.5 at the cardiomyocyte perinexus [37]. The relationship between Cx43 and Na_v_1.5 might help to explain the theory of electrical field (or ephaptic) propagation in cardiac tissue. The classical view on impulse propagation is based on the principles contained in continuous cable theory, representing gap junctions as passive resistors providing the only pathway for the passage of charge between cardiomyocytes. This theory predicts that a reduction in gap junctional communication should be accompanied by a reduction in conduction velocity. However, this has been demonstrated to not always be the case. Impulse propagation has been shown to possess a high safety factor, and conduction velocity is only moderately reduced after loss of more than 90% of gap junctional channels [256,257,258,259]. In electrical field theory, the large inward sodium current in the proximal side of an intercellular cleft would generate a large negative extracellular potential within the cleft, thus depolarizing the sarcolemma of the distal cell. This would result in activation of its sodium channels, allowing downstream impulse propagation [260,261,262,263,264]. Although this mechanism would be almost insignificant when gap junctions are opened, it may be crucial to maintain conduction when gap junctions are closed or connexin expression is reduced [249,264]. However, although ephaptic propagation has been simulated theoretically under very specific conditions, it has never been proven experimentally [262].

#### 2.5.4. Other Interactors of Sarcolemmal Connexins

Cx43 has also been shown to colocalize and coimmunoprecipitate with both caveolin-1 and -2 [265,266], structural membrane proteins acting as scaffolds to cluster lipids and signaling molecules within the caveolae [265]. The interaction of Cx43 with caveolin-1 seems to be direct and to occur at the CT domain of the former [265,266], whereas binding of caveolin-2 seems indirect, through caveolin-1 [266]. In rat epidermal keratinocytes, newly synthetized Cx43 interacts with caveolins at the Golgi apparatus and both proteins are trafficked together to lipid rafts within the plasma membrane [266]. Cx43 appears, then, to dissociate from caveolins and lipid rafts prior to gap junction assembly [266]. Although caveolins are not essential for Cx43 transport to the cell membrane, this interaction may constitute an additional mechanism to regulate gap junctional intercellular communication [266]. In addition, the interaction of Cx43 with caveolins may facilitate regulation of Cx43 by PKCγ within the caveolae, as demonstrated in epithelial cells after PKC activation by TPA or IGF-1, which results in a decrease in the gap junction plaque size [267].

A number of additional protein partners of connexins have been identified [144,225,226], including several regulators of cell growth, differentiation and migration [8]. β-catenin, in addition to being part of anchoring junctions, is a transcriptional coactivator able to translocate from the cytoplasm to the nucleus, and to regulate cell proliferation and differentiation. It has been proposed that Cx43 may negatively regulate its own transcription, in addition to modulating the expression of several other β-catenin targets, by sequestering it at the cell membrane [268]. On the other hand, interaction with CNN3, a member of the CNN (Cyr61/connective tissue growth factor/nephroblastoma-overexpressed) family of proteins, may mediate the inhibitory effect of Cx43 in cell growth and the enhancement in cell adhesion, both actions being independent of gap junctional communication [269,270]. Interaction of MAPK-phosphorylated Cx43 and cyclin E has been demonstrated to be important in vascular smooth muscle cell proliferation [271]. In addition, it has been shown that the molecular chaperone heat shock cognate protein 70 (Hsc70) directly binds to the CT domain of Cx43, thus competing with cyclin D1 for its binding to Hsc70, counteracting its translocation to the nucleus and modulating cell proliferation and cell cycle progression [272]. Finally, Cx43 has also been proposed to sequester β-arrestin in osteoblasts, thus reducing its association with the parathyroid hormone receptor, facilitating cAMP signaling and exerting an anti-apoptotic effect [273]. However, these channel-independent effects of connexins, which are dependent on modulation of the activity of their interacting partners, have not been investigated in cardiac tissues.

Taken together, the present evidence suggests that the intercalated disc is not simply composed of independent molecules, with each one having their own functions, but rather behaves as a complex organelle that involves multiple interactions. These interactions are relevant to excitability, propagation and mechanical coupling between neighboring cells, but they go beyond these functions and extend to regulation of cell growth and differentiation, among others.

#### 2.5.5. Interactions of Mitochondrial Connexins

**Figure 6:**
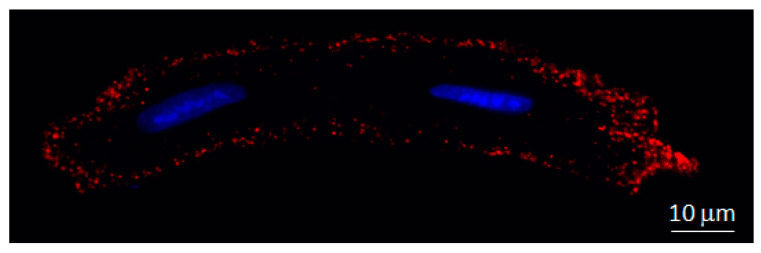
Confocal fluorescent image of a proximity ligation assay (PLA; Duolink) showing interaction of Cx43 with mitochondrial respiratory complex II at subsarcolemmal mitochondria. Nuclei were stained with Hoechst 33642 (blue).

Cx43 at cardiomyocyte mitochondria has been shown to immunoprecipitate and/or colocalize with proteins involved in its translocation to the inner mitochondrial membrane, including Tom20, a member of the TOM/TIM (translocase of the outer membrane/translocase of the inner membrane) import system, and with heat shock protein 90 (Hsp90) [24], but also with others that are essential for mitochondrial function, such as ANT (adenine nucleotide transporter) or the mitochondrial respiratory complex II (Figure 6) [24,25]. In addition, it interacts with apoptosis-inducing factor (AIF) and with the beta subunit of the electron transfer protein (ETFB), two proteins involved in oxidative phosphorylation and redox control [274]. Finally, Cx43 has been suggested to also interact with Bax, a protein that accumulates at the outer mitochondrial membrane forming pores that mediate apoptosis [275], and with specific subunits of mitochondrial ATP-dependent K^+^ (mitoK_ATP_) channels [276], which are involved in some forms of protection. The interaction with Bax occurs at the Cx43 CT domain, following its translocation to the mitochondria, and may be, at least in part, responsible for the tumor-suppressive effects of Cx43 in pancreatic cancer cells [275].

## 3. Cardiac Connexins

GJIC is particularly relevant in the mammalian heart. It was in this organelle where the existence of direct intercellular communication between adjacent cells was first proposed, in 1925 [277]. The earliest images of vertebrate gap junctions, appearing as pentalaminar structures in cross-sectional images, were obtained from mouse and guinea pig hearts and were published in the late 1950s [278]. The name “gap junction” was coined in 1967 by Revel and Karnovsky, based on observations made in ventricular cardiac tissues demonstrating that, in these areas, the plasma membranes of two adjacent cells were in close contact (20–30 Å), but not fused, and that heavy metals such as lanthanum could be infused into the space or “gap” between both membranes [43]. Later on, freeze-fracture electron microscopy studies demonstrated that gap junctions contain packed arrays of intercellular channels, which are arranged in a lattice of hexagonal structures [43,279].

Most cell types within the myocardium, including cardiomyocytes, fibroblasts, leukocytes and endothelial and smooth muscle cells, express connexins [280,281]. However, here, we are going to refer mainly to those expressed in cardiomyocytes. Although they represent less than a third of the total cell number in the heart [282,283,284], cardiomyocytes occupy 70–85% of the total cardiac volume and are responsible for maintaining the cardiac beat. Cardiomyocytes express three main connexin isoforms, which are essential to sustain a coordinated cardiac contraction: Cx43, Cx40 and Cx45 [281,285,286,287]. However, Cx43 is, by far, the most abundant connexin isoform expressed in the heart [288]. Although its specific location depends on the animal species, in general, it can be considered that Cx43 is widely expressed both in the ventricular myocardium and in the atria, and in some parts of the conducting system [281,286,288]. Initial studies conducted in rat hearts demonstrated that Cx43 expression is increased during embryonic development, which correlates with an enhancement in conduction velocity [288]. On the other hand, Cx40 can be found in the atria, the atrioventricular node and in the His–Purkinje system [281,285,286]. Interestingly, the distribution patterns of Cx40 and Cx43 are, to a large extent, comparable in rat, guinea pig, porcine, bovine and human hearts, both in neonates and adults [5,285,286,287,289]. Regarding Cx45, its expression is mostly restricted to the pacemaker and conducting system [5,281,285,290], representing only about 0.3% of the total connexin content in the ventricular myocardium [200]. An additional connexin isoform, Cx30.2, is also present in the mouse sinoatrial node and other parts of the conducting system, although this might be a special rodent feature [285,291]. In fact, Cx31.9, the human orthologue of mouse Cx30.2, is not detectable in the human cardiac conduction system [292]. Finally, Cx46 has been reported to also be expressed in the murine conducting system [293].

As it has been discussed previously, connexins are predominantly expressed at both cardiomyocyte ends, forming plaques of intercellular channels that put into contact the cytoplasms of adjacent cells (Figure 2) [13]. These plaques, the gap junctions, are one of the components of the intercalated disc, a complex cell structure in which plasma membranes of neighbouring cells are in close contact, and that also includes adherens junctions, desmosomes and other ion channels [14,15]. In the heart, Cx43 located at gap junctions is predominantly found, under normal conditions, in the two slower-migrating forms, P2 and P1 (Figure 5) [23,24,42,149,258,294,295,296,297]. In contrast, the amount of the faster-migrating, non-phosphorylated Cx43 species (P0) is often markedly lower or even below the detection limit (Figure 5) [23,24,42,149,258,294,295,296,297].

### 3.1. Alterations in Connexin Distribution and Phosphorylation under Pathological Conditions

Both the normal distribution of connexins and their phosphorylation state are often disturbed under pathological conditions. This would be the case of myocardial ischemia, where gap junction remodeling occurs rapidly after coronary occlusion (Figure 5) [298,299]. Redistribution of gap junctions outside intercalated discs was first reported in the ventricular myocardium bordering the infarct scar in patients with advanced ischemic heart disease [300]. This was later confirmed in the epicardial border zone of healing myocardial infarctions in dogs, where Cx43 was shown to be mainly distributed, 4 days after coronary occlusion, along the lateral surface of cardiomyocytes [301]. Importantly, these areas of disturbed distribution correlated with the location of reentrant circuits causing ventricular tachycardia [301]. Studies in experimental models have allowed demonstrating that periods of ischemia as short as 15 min are able to induce gap junctional remodeling to the lateral membranes of cardiomyocytes [149,302,303,304]. Importantly, electron microscopy analysis revealed that both true gap junctions connecting side-by-side myocytes and vesicles containing internalized Cx43 contribute to this lateral signal [300]. True lateralized gap junctions have been previously suggested to be fully functional [305], whereas transfer of Cx43 to intracellular pools has been demonstrated to occur during ischemia [296,306].

The shift of Cx43 from the intercalated disc to the lateral membranes of cardiomyocytes is referred to as lateralization [42] and is often accompanied by changes in phosphorylation (Figure 5). Ischemia induces a progressive dephosphorylation of Cx43, with a shift to the faster migrating species in SDS-PAGE occurring within minutes (Figure 5) [296,304,306,307,308,309,310,311]. Specifically, Cx43 has been shown to be dephosphorylated during ischemia at Ser279, Ser282, Ser325/328/330 and Ser365, while phosphorylations at Ser262, Ser373 and Ser368 are increased [42,149,304,309,310,312]. Furthermore, Cx43 remaining within the intercalated discs following myocardial ischemia seems to be in the phosphorylated state, whereas that in the lateral membranes has been suggested to be mostly dephosphorylated [149]. Interestingly, Cx43 dephosphorylation has been associated with the onset of cell-to-cell electrical uncoupling [296]. Less clear are, however, the effects of ischemia on the total amount of Cx43. Thus, whereas some authors have suggested that myocardial ischemia induces a progressive reduction in total Cx43 in different experimental models [149,296,302,308,311], others have not been able to demonstrate such decrease in expression [306,307,310].

The acute changes in Cx43 expression and phosphorylation occurring shortly after ischemia might be modified as the healing process progresses. Analysis of canine healed infarcted hearts, performed 3–10 weeks after coronary occlusion, demonstrated fewer side-to-side connections between myocytes located at the infarct border zone [313]. This change was accompanied by a reduction in the size and number of gap junction plaques at intercalated discs. Together, both alterations would, potentially, enhance the risk of reentrant arrhythmias [313].

Gap junction remodeling has been shown to also occur under other pathological conditions. Thus, a progressive and heterogeneous reduction in Cx43 levels, often accompanied by enhanced lateralization, has been described in myocardial samples from patients with heart failure of different etiologies, including those secondary to ischemic, dilated and inflammatory cardiomyopathy [314,315,316,317]. Similar findings have been obtained in patients with systemic or pulmonary hypertension [315,318], a disorder also associated with reduced Cx43 phosphorylation [319]. Interestingly, the downregulation of cardiac Cx43 expression and the decreased size of gap junctional plaques observed in patients with congestive heart failure have been related to enhanced colocalization with zonula occludens-1, a protein that regulates, as discussed before, the gap junction size and endocytosis [320]. Similarly, enhanced dephosphorylation of Cx43 and translocation from the intercalated discs to intracellular pools have been demonstrated in a rat model of ventricular hypertrophy induced with monocrotaline [321]. Furthermore, enhanced formation of annular gap junctions was detected in this last model, suggesting accelerated gap junction degradation [321]. Regarding diabetes, it has been suggested that it may induce heterogeneous changes within the myocardium in rats treated with streptozotocin, including gap junction lateralization, reduction in the number of gap junctions at intercalated discs and enhanced total Cx43 expression [322,323], changes associated with increased vulnerability to hypokalemia-induced ventricular fibrillation [323]. Contrarily, however, others were not able to confirm these findings in diabetic rabbits [324]. Finally, and although this is not a cardiac disease per se, aging also alters gap junction organization. Thus, hearts from aged animals have been shown to depict a reduction in Cx43 levels together with some degree of lateralization [325].

In addition to these acquired diseases, inherited cardiomyopathies have also been associated with redistribution or changes in phosphorylation of Cx43. This is the case of oculodentodigital dysplasia (ODDD), an autosomal dominant disease characterized by developmental abnormalities in limbs, teeth, face and eyes, due to mutations in the GJA1 gene, encoding Cx43 [326]. Thus, a murine model of ODDD carrying the disease-causing point mutation I130T, located at the cytoplasmic loop domain, depicted a reduction in Cx43 at gap junctions, with preferential loss of phosphorylated isoforms [327,328]. A similar loss of the P2 phosphorylation state was described for the G138R point mutation, which also resides within the cytoplasmic loop [328]. These data were consistent with a dominant negative effect of the mutant protein on the stability of the wild-type allele [327]. On the other hand, arrhythmogenic right ventricular cardiomyopathy, a disease mostly caused by mutations in genes encoding desmosome proteins, including plakophilin-2, plakoglobin, desmoglein-2 and desmoplakin [329], has been shown to be associated with secondary effects in Cx43 expression. Indeed, the majority of cases are associated with reductions in the amount of plakoglobin at the intercalated discs, but also with a decrease in the size and number of Cx43-formed gap junctions [262,330,331]. Reductions in both plakoglobin and Cx43 at the intercalated discs seem to be independent of changes in the total amount of both proteins, which may be suggestive of a defect in protein trafficking [262]. Furthermore, studies in cardiomyocyte-specific desmoplakin-deficient mice, a model of arrhythmogenic right ventricular cardiomyopathy, confirmed a primary loss of Cx43 expression, phosphorylation and function, effects associated with enhanced vulnerability to ventricular arrhythmias [332]. Other changes occurring in patients with arrhythmogenic right ventricular cardiomyopathy include a reduction in Na_v_1.5 sodium channels [262], which may indicate a global alteration of the connexin interactome.

Despite the fact that the pathophysiology of atrial fibrillation is complex, and probably multifactorial, the disease has often been linked to changes in the expression and/or distribution of the main atrial connexin, Cx40 [281]. It has been demonstrated that Cx40 expression becomes heterogeneous and redistributes to the lateral sides of cardiomyocytes in human atrial samples from patients with atrial fibrillation [333,334,335,336]. A similar lateralization has also been described for Cx43 [333,334]. However, contradictory data have been published regarding changes in total Cx40 expression. Thus, whereas Western blot analyses of samples from patients with paroxismal or chronic atrial fibrillation have demonstrated a reduction in Cx40 levels [336], others have described no changes [337], or even an increased amount of the protein in patients with ischemic heart disease undergoing coronary artery bypass surgery that subsequently developed atrial fibrillation [334] or in patients with chronic atrial fibrillation [333]. No clear explanation is available for these discrepancies, but they may rely on the multifactorial origin of the disease or on differences in the exact location of the analyzed tissue within the atria [335]. In addition, induction of persistent atrial fibrillation by burst pacing in goats leads to heterogenous distribution of Cx40 [338]. On the other hand, total expression of Cx43 seems not to be modified by the disease [333,334].

Mislocation of connexins and gap junctions outside intercalated discs and modifications in their phosphorylation state result in important alterations in cardiomyocyte electrical coupling, thus disturbing action potential propagation and contributing to arrhythmia susceptibility [301,313,323]. Furthermore, such changes may also modify the connexin interactome, altering trafficking of other proteins, including Na_v_1.5 sodium channels [245], which would result in changes in cell excitability. Considering the points discussed above, connexin remodeling may have important implications in cardiac pathophysiology.

### 3.2. Functions of Cardiac Connexins

The main functions of connexins are related to their ability to form gap junctional intercellular channels. However, the way we understand these proteins has dramatically changed in recent years. In addition to these widely known roles, connexins exert other less conventional functions, which are independent of their ability to form gap junctional channels. These non-canonical functions of connexins include, as we will discuss below, those ascribed to unopposed hemichannels, to mitochondrial connexins and to the N-terminally truncated isoforms, a series of fragments derived from the CT domain of the molecule.

#### 3.2.1. Sarcolemmal Connexins: Gap Junction-Dependent Role in Cardiac Electrical Coupling

The heart is composed of billions of individual cardiac myocytes but functions as a whole, i.e., as a highly coordinated syncytium. Such precise coordination, needed to maintain a proper cardiac function, is based on the fact that individual cardiomyocytes contract in synchrony, in a process that is dependent on rapid propagation of electrical excitation. It was in 1952 when Weidmann, applying classical electrical cable theory, was able to demonstrate that an electrical current injected into a single cell in a Purkinje strand was able to propagate along a distance larger than the length of the cell itself [339]. This finding supported the hypothesis that electrical charges move freely from one cardiomyocyte to the next, thanks to the existence of low-resistance pathways between them [339]. Indeed, the existence of these paths of low resistance was later confirmed in ventricular muscle [340], and they are nothing more than these aggregations of intercellular channels that we now know as gap junctions. Gap junction channels bridge the cytoplasm of neighboring cells, thus forming cell-to-cell pathways that allow rapid electrical current flow between them. Nowadays, their importance for cardiac function and electrical coupling is beyond any doubt [134,262,263,341,342].

##### The Cable Theory of Electrical Conduction and the Influence of Tissue Anisotropy

Linear cable theory predicted an inverse relationship between conduction velocity and the square root of axial tissue resistance, as described by the formula θ = k/$\sqrt{r_{i}+r_{e}}$, where θ represents conduction velocity and *r_i_* and *r_e_* are the intracellular and extracellular resistances, respectively [263,343,344]. To reconcile the existence of gap junctions with the apparent continuous conduction, gap junctional resistance should be sufficiently low to render the cytoplasms of coupled cells contiguous [340,345], and, consequently, gap junctional resistance is usually included within intracellular resistance [344]. Indeed, the predicted relationship between conduction velocity and axial resistance was consistent with experimental measurements conducted in the late 1950s [346,347].

Cardiac muscle is an anisotropic structure, whose components are individual cardiomyocytes that are 100–150 µm long and 10–20 µm wide, which are end-to-end connected by gap junctions (Figure 2). This cardiac architecture would cause the direction of the impulse spread to determine cardiac conduction. Thus, early works in the late 1950s demonstrated that conduction velocity in the longitudinal direction is larger than that in the transversal direction [343,344,347]. This finding was later confirmed by other authors [256,348,349,350]. Continuous cable theory, which was initially applied to understand action potential propagation in nerves and later adapted to Purkinje fibers, was then updated, in order to incorporate the anisotropic cardiac structure into two- and three-dimensional myocardial models, allowing one to confirm that, indeed, conduction velocity in cardiac tissue is anisotropic [351,352].

Later on, a series of experiments analyzing conduction velocity and action potential upstroke at high spatial and temporal resolutions allowed demonstrating that, in fact, cardiac conduction is not continuous but rather discontinuous at the microscopic level. Cable theory predicts that the shape of the action potential foot, before the fast upstroke, should depend only on axial and membrane resistances, but not on conduction velocity, and that the maximal rate of the rise in the transmembrane action potential (dV/dt_max_) should depend only on Na_v_1.5 channel availability (i.e., a faster conduction velocity should be associated with a larger dV/dt_max_). However, studies in both the atrial and the ventricular myocardium demonstrated that faster longitudinal conduction was associated with longer foots and a smaller dV/dt_max_, whereas the contrary was true for the slower transverse conduction [353,354]. In those studies, gap junctions may represent recurrent discontinuities in axial resistance, causing propagation to be discontinuous on a microscopic scale [353,354]. In fact, results of computer simulations suggested that propagation is actually saltatory, based on rapid excitation of individual cells followed by a conduction delay at gap junctions [355,356,357]. Indeed, it was revealed that gap junctions may represent high-resistance pathways at a microscopic scale, with a resistance similar to the axial resistance of the rest of the myocyte [355]. Under these conditions, membrane capacitance should be charged before conduction progresses in the distal direction. Although these experiments questioned the use of models based on continuous cable theory to describe anisotropic conduction, it should be taken into account that impulse propagation is saltatory under physiological conditions along single-cell chains. However, this feature might be attenuated in intact multicellular tissues, due to the effect of lateral gap junctional coupling that averages small differences in activation times of individual cardiomyocytes [355]. In these three-dimensional tissues, saltatory conduction would appear only under critical gap junctional uncoupling [355].

##### Gap Junctions and Electrical Coupling in the Supraventricular Conduction System

Pacemaker activity in the sinoatrial node initiates normal cardiac beats, which are conducted along the atria to the atrioventricular node. Upon arrival to it, activation of the entire ventricular myocardium occurs via the specialized conduction system. Impulse propagation depends on three main factors, including excitability of single cardiomyocytes, electrical coupling between them and the network properties of the cardiac tissue [342], in a process that is also greatly influenced by cell size [358]. While cell excitability is determined by the amount of inward currents I_Na_ and I_Ca_, electrical coupling is dependent on cell-to-cell communication through gap junctions [134,262,263,341,342].

The two main functions of gap junctions at the sinoatrial node are the maintenance of beating at a regular frequency, and the transmission of the impulse to the atrial myocardium [342]. However, both functions need only a small amount of coupling [359]. Immunohistochemical studies have demonstrated that Cx45 is the predominant isoform at the node center, but some controversy exists regarding the presence of other connexins at the nodal periphery [360]. Interestingly, it has been hypothesized that heterotypic gap junctions, formed by one Cx45 connexon and one Cx43 hemichannel, may form at the periphery between sinoatrial and atrial cells, respectively. In this way, these heterotypic gap junctions would close when the current flows from the atria to the node but would open when the current flows from the node to the atria [259].

Two main connexin isoforms are expressed in the atria, Cx43 and Cx40 [281,285,286,342]. However, most studies point to a predominant role of the latter in sustaining atrial conduction. Thus, homozygous Cx40 knock-out mice were shown to depict a reduced atrial conduction velocity, a finding that was associated with P wave prolongation in the electrocardiogram [361]. Moreover, Cx40 knock-out mice are more vulnerable to supraventricular tachyarrhythmias [361,362], whereas polymorphisms in the Cx40 promoter are associated with enhanced atrial vulnerability and increased arrhythmia susceptibility [363]. On the other hand, conditional knock-out mice for Cx43 did not depict any effect on the P wave duration or atrial conduction velocity, suggesting that Cx43 is not the main connexin isoform responsible for atrial conduction in the presence of Cx40 [364]. Interestingly, treatment with carbenoxolone, a gap junction uncoupler derived from glycyrrhetinic acid, has been shown to depress right atrial conduction in patients undergoing electrophysiology studies [365], confirming the importance of connexins in atrial conduction. On the other hand, an enhancement in gap junctional coupling by treatment with rotigaptide (ZP123) or danegaptide (GAP-134) has been associated with improvements in atrial conduction and with reductions in the incidence of atrial fibrillation in different experimental models [366,367,368]. The mechanisms of action of these derivatives of antiarrhythmic peptide (AAP), which was originally isolated from bovine atria [369,370], are not well understood, but they are believed to enhance gap junctional communication through modifications in the phosphorylation state of connexins [179], independently of changes in other ionic channels [371,372].

Three connexin isoforms have been described in the atrioventricular node of mice, Cx40, Cx30.2 and Cx45 [281,285,286,342]. The absence of Cx40 has been consistently associated with abnormal atrioventricular conduction, as demonstrated by prolonged PQ intervals in electrocardiogram recordings [361,373]. On the contrary, heterozygous Cx45 deficiency did not modify atrioventricular conduction, although full deletion was not investigated due to its lethality [373]. However, in the absence of Cx40, Cx45 haplodeficiency further worsened the atrioventricular conduction delay [373], suggesting some type of interaction between both connexin isoforms.

##### Gap Junctions and Electrical Coupling in the Ventricular Myocardium

Cx43 is the main connexin isoform expressed in the working ventricular myocardium. In fact, Cx43 has been the most studied isoform with regard to its role in cardiac conduction. The involvement of Cx43 and gap junctions in ventricular conduction has been supported by computer simulations of microscopic impulse propagation. In those studies, a reduction in gap junctional conductance resulted in a decreased conduction velocity, in addition to accentuated saltatory behavior [355,356,357,374,375,376]. Furthermore, pharmacological blockade of gap junctional channels with octanol or palmitoleic acid reduced ventricular conduction velocity in cell strands from neonatal rat hearts [377], whereas treatment with octanol in guinea pig papillary muscles resulted in slower propagation of action potentials before complete conduction block [357]. In addition, both heptanol and 18α-glycyrrhetinic acid, two classical gap junction uncouplers, were shown to induce a concentration-dependent depression of conduction in normoxic isolated rat hearts [378]. Similar results were obtained with a variety of gap junction uncouplers in other experimental models, including mice, rabbit and guinea pig isolated hearts, and in canine and sheep epicardial ventricular slices [379,380,381,382,383,384,385]. In addition, carbenoxolone depressed ventricular conduction in patients undergoing electrophysiology studies [365]. Importantly, the effects of gap junction uncouplers were often accompanied by enhanced anisotropy [381,382,386] and arrhythmogenesis [385,387].

However, results obtained in transgenic mice models are conflicting. Cx43 knock-out (Cx43^−/−^) mice die soon after birth, due to cardiac malformations of the pulmonary outflow tract [388]. For this reason, studies analyzing the role of Cx43 on ventricular conduction were initially conducted in heterozygous Cx43 knock-out (Cx43^+/−^) mice. Thus, early studies suggested that ventricular epicardial conduction of paced beats is slower in hearts from neonatal heterozygous Cx43^+/−^ mice, as compared with hearts from wild-type animals [389]. This phenotype was even more severe in adult mice, a finding that was associated with prolongation of the QRS complex in the electrocardiogram, but not with differences in action potential characteristics [389]. These results were later confirmed by studies using high-resolution optical mapping of ventricular action potentials at the epicardial surface of Langendorff perfused Cx43^+/^^−^ mice hearts [349,364]. In contrast, others have not found differences in electrocardiogram measurements, including the QRS duration, between Cx43^+/^^−^ and wild-type mice, and both groups of animals depicted similar epicardial activation patterns and conduction velocities [256].

In order to circumvent perinatal lethality associated with germline inactivation of the Cx43 gene, a cardiac-restriced knock-out mice model was later developed [350]. In these animals, the Cre/loxP system was used to ablate Cx43 expression exclusively in cardiomyocytes, with Cre activity being driven by regulatory elements from the α-MHC gene. They depicted a marked and progressive reduction in Cx43 expression as early as embryonic days 9.5–12.5, which caused a significant prolongation in the QRS complex duration, together with a decrease of up to 42% in conduction velocity in the longitudinal direction and 55% in the transversal direction, and an increase in the anisotropic ratio, as compared with their control littermates [350,390]. However, when these animals were selectively bred to slow down the progressive reduction in Cx43 expression, the authors found that at 25 days of age, when Cx43 levels were about 59% of control values, conduction velocity, assessed by optical mapping in isolated hearts, was not significantly altered [257]. It was necessary to reduce Cx43 to about 18% of the normal content (at 45 days of age) to slow down the ventricular conduction velocity to about half of that observed in control hearts [257]. Similar results were obtained in an inducible knock-out Cx43^Cre-ER(T)/fl^ mice model, in which a global ablation of Cx43 was achieved after 4-hydroxytamoxifen (4-OHT) administration [391,392,393]. In this model, Cx43^Cre-ER(T)/fl^ animals treated with vehicle, having about 50% of the normal Cx43 content, did not depict any change in epicardial activation patterns, activation delays or conduction velocity [258,391,392]. In contrast, treatment of these mice with 4-OHT induced a marked decrease in Cx43 expression, above 95%, 14 days after induction, and this effect was associated with QRS complex prolongation, a reduction in conduction velocity, especially in the transversal direction, and enhanced anisotropy [258,391,392,393]. In the same line, replacement of Cx43 with Cx32, a connexin isoform with only a slightly lower conductivity and permeability, in Cx43KI32 mice, did not affect ventricular conduction and induced only minor changes in electrocardiographic variables [394]. Taken together, these findings may indicate that a marked reduction in Cx43 expression, over 90%, is needed to obtain noticeable effects in conduction velocity.

In relation to studies analyzing the effects of connexin deficiency on ventricular conduction velocity, results regarding the incidence of ventricular arrhythmias point to the same direction. Thus, reduced Cx43 expression in Cx43^+/−^ mice has been associated with accelerated onset and increased incidence and duration of ventricular tachyarrhythmias after coronary occlusion in isolated hearts [395]. Contrarily, others have not been able to demonstrate an increased occurrence of either spontaneous or inducible ventricular tachyarrhythmias in the same animal model, 6 days or 10 weeks after myocardial infarction [396]. On the other hand, telemetry recordings from mice with cardiomyocyte-restricted inactivation of Cx43 showed a high incidence of spontaneous and induced ventricular tachyarrhythmias under baseline conditions, which could not be terminated by high-frequency pacing, whereas their control littermates had no arrhythmic events [350,397]. Furthermore, these animals developed sudden death due to spontaneous ventricular tachyarrhythmias at 2 months of age [350]. However, and similar to conduction velocity, selective breeding of these animals to slow down the decrease in Cx43 levels allowed demonstrating that ventricular tachyarrhythmias were only inducible when Cx43 expression was markedly reduced to less than 18% of that present in normal hearts, at 45 days of age [257,398]. Further supporting that a marked reduction in Cx43 expression is needed to observe noticeable effects in arrhythmogenesis, programmed electrical stimulation resulted in ventricular arrhythmias only in Cx43^Cre-ER(T)/fl^ animals treated with 4-OHT (having less than 95% of normal Cx43 expression 14 days after induction), but not in those treated with vehicle (50% of the normal Cx43 content) [391]. In fact, animals treated with 4-OHT had about 50% of mortality 14 days after induction, and telemetric recordings allowed demonstrating the occurrence of polimorphic ventricular tachyarrhythmias just before death [393]. A similar enhancement in arrhythmogenesis was obtained in isolated mice hearts from the same animal model after 4-OHT, both under normoxic conditions and during ischemia/reperfusion [258]. The enhanced arrhythmogenesis seen in isolated hearts from Cx43KI32 mice, both under normoxic conditions and during ischemia/reperfusion, despite the fact these hearts had no apparent effects in conduction, might be explained by differences in connexin regulation between both isoforms [258].

This behavior, in which ventricular conduction is depressed only after a marked reduction in Cx43 expression, can be explained by the fact that cardiac conduction has a high safety factor [262,355,356]. The safety factor is the ratio between the electrical charge produced by an excited cell and the charge needed to depolarize it, with propagation being successful when this value is higher than 1 [262]. A reduction in gap junctional coupling will reduce the amount of axial current exciting downstream cells, thus theoretically decreasing conduction velocity. However, it would, simultaneously, enhance the safety factor, as less current would be dissipated into the following cell [262,356]. This would result in a faster charge of the membrane capacitance and, thus, a faster dV/dt_max_, which may even result, under some circumstances, in a paradoxical improvement in impulse conduction [399]. As a consequence, conduction can be maintained at very low velocities, even at low levels of intercellular coupling [262,356]. Only when intercellular coupling is markedly reduced would the safety factor decrease and conduction block occur [262,356].

The importance of Cx43 in ventricular conduction and arrhythmogenesis has been strengthened by the finding that an enhancement in gap junctional coupling by antiarrhythmic peptides, including AAP10 and rotigaptide, two AAP derivatives, increased ventricular conduction under baseline conditions and prevented conduction slowing during ischemia in isolated rabbit and guinea pig hearts [400,401]. Furthermore, both rotigaptide and danegaptide have been shown to attenuate the incidence of ventricular arrhythmias in dogs submitted to transient coronary occlusion [402,403], whereas AAP10 prevents development of torsade de pointes (TdP) in long QT syndrome animal models [404,405]. Similarly, an enhancement in gap junctional communication by adenoviral-mediated Cx43 gene transfer in pigs induced faster conduction velocities in the anterior septal border, and treated animals were less prone to develop inducible ventricular tachyarrhythmias after coronary artery occlusion [406].

##### Gap Junctions and Ischemic Ib Ventricular Arrhythmias

Gap junctional uncoupling has been suggested to be of great relevance during myocardial ischemia, as it might be involved in the genesis of ischemic Ib ventricular arrhythmias. Following an ischemia, ventricular arrhythmias appear grouped in two different episodes, both having different mechanisms [407,408]. Phase I arrhythmias appear almost immediately after coronary occlusion and last for about 30–40 min, whereas phase II or subacute arrhythmias begin several hours later and last for about 24–48 h. Phase I arrhythmias can be subdivided into phase Ia (first 2–10 min of ischemia) and phase Ib (from 10 to 40 min) [408,409]. Importantly, phase Ib ventricular arrhythmias have been related to the appearance of ventricular fibrillation, with an increase in intracellular resistance denoting gap junction closure, as determined in the cable-like rabbit papillary muscle. In these studies, myocardial tissue was electrically simulated by a circuit composed of three main elements: intracellular (r_i_, cytosol plus gap junctions) and extracellular (r_o_) resistances, connected in parallel, and a membrane capacitance between them [410]. Interestingly, the cable-like rabbit papillary muscle preparation allowed a separate analysis of both intracellular and extracellular resistances [411,412,413,414]. Using this experimental setup, it was possible to demonstrate that during the first 10–15 min of ischemia, r_o_ increases, while r_i_ remains relatively constant. The increase in r_i_ occurs thereafter and may reflect cell-to-cell electrical uncoupling, as it was associated with a reduction in conduction velocity [411,412,413,415,416,417]. Later, measurements of myocardial electrical impedance, using the four-electrode method, confirmed these findings [84,296,418,419,420,421]. This technique allowed demonstrating that coronary occlusion is associated with an initial slight increase in total tissue resistance, probably due to changes in r_o_, that is followed by an abrupt second increase, which would correspond to the enhancement in r_i_ [421,422,423,424]. The onset of the abrupt increase in r_i_ is closely linked to the development of rigor contracture, which reflects a critical reduction in ATP levels, with the rise in cytosolic Ca^2+^ concentrations [415,416], and always preceded the appearance of phase Ib of ischemia-induced ventricular arrhythmias [421,422]. In fact, it was demonstrated that the onset of cell-to-cell electrical uncoupling, as assessed by the four-electrode technique in open-chest swine, positively correlated with the occurrence of ventricular fibrillation during such phase [421]. Furthermore, ischemic preconditioning, a cardioprotective maneuver consisting in brief cycles of ischemia/reperfusion applied before a more prolonged, and potentially lethal, ischemic episode, has been shown to delay both cell-to-cell uncoupling and phase Ib arrhythmias [422]. Taken together, these findings suggest that loss of cell-to-cell communication might be involved in the genesis of phase Ib arrhythmias during myocardial ischemia.

#### 3.2.2. Sarcolemmal Connexins: Gap Junction-Dependent Role in Chemical Coupling

As discussed in previous sections, connexins are permeable to a wide variety of ions and second messengers [76,77,78,79,80,81,82], allowing the existence of chemical coupling between neighboring cells. Longitudinal diffusion experiments of radioactive potassium, carried out in 1966 in bundles of sheep ventricular fibers, allowed demonstrating free diffusion of this ion through intercalated discs [340]. Since these initial studies, the permeability of connexin channels has been extended to Cl^−^ and Cs^+^ [75], Na^+^ [425], Ca^2+^ [81], ATP and ADP [83,426], glutathione and glutamate [426], IP3 [81,427], AMP and adenosine [83], cAMP [428,429] and glucose [82], among many others. It is generally assumed that gap junctional channels are permeable to solutes with a molecular weight below 1.5 kDa, with selectivity depending on the exact connexin isoform. Moreover, permeability is not only affected by size but also by the net charge of the molecule [79].

##### Involvement in Myocardial Ischemia/Reperfusion Injury

The role of chemical coupling through gap junctions in cardiac physiology has not been properly established. However, it is well known that it participates in the propagation of intracellular calcium waves, probably through diffusion of cytosolic messengers such as IP3, in a number of tissues, including endothelial, astrocytic and osteoblastic cells [430,431,432,433]. Furthermore, it is now apparent that it may also play a key role under some pathological conditions, including cardiac ischemia/reperfusion injury [84]. Myocardial ischemia is associated with increased cytosolic Ca^2+^ concentrations, reduced ATP levels and tissue acidosis, factors inducing cardiomyocyte gap junction closure [122,434,435]. These changes closely correlated with the onsets of ischemic rigor contracture and cell-to-cell electrical uncoupling, as we discussed earlier [415]. However, gap junction closure, which was initially associated with the “healing over” phenomenon, is not an immediate process. Instead, it develops progressively during the ischemic insult, as denoted by analysis of tissue electrical impedance in the entire myocardium and of intracellular resistance in rabbit papillary muscle preparations [411,412,415,416,418,422,423,424]. Furthermore, reperfusion is associated with a quick recovery of tissue acidosis and ATP levels, thus allowing re-opening of closed gap junctions, as demonstrated by restoration of initial resistivity values in tissue impedance analysis [423,424].

Under these conditions of progressive closure of gap junctional channels during ischemia and re-opening during reperfusion, residual communication may allow spreading of the intracellular derangements caused by ischemia/reperfusion [84]. Indeed, computer simulations performed in the late 1980s demonstrated that the only means to reproduce the infarct geometry was to enter into the software a contiguity condition requiring direct contact between irreversibly injured cells [436]. In fact, histological analyses showed that dead, hypercontracted cardiomyocytes are not found scattered across the area at risk. Instead, they appear connected to each other in specific areas known as contraction band necrosis [436]. These initial studies allowed suggesting that some kind of physical interaction between cardiomyocytes was an important determinant of progression of necrosis during coronary occlusion. Later, this interaction was shown to be chemical, to occur mainly during reperfusion and to be mediated through gap junctions. Thus, the gap junction uncoupler heptanol was able to prevent cell-to-cell progression of hypercontracture and, when given at the onset of reperfusion, modified the infarct geometry and reduced the extent of contraction band necrosis and infarct size [437]. These findings were later confirmed by studies using other chemically unrelated gap junction uncouplers, including 18α-glycyrrhetinic acid, carbenoxolone, halothane and palmitoleic acid [309,378]. Furthermore, protection by gap junction uncouplers was associated with attenuated recovery of myocardial electrical resistivity during reperfusion, which was suggestive of reduced gap junction re-opening [378]. The mechanisms by which gap junction closure protects against propagation of hypercontracture and cell death include passage of Na^+^ from injured to healthy cells, and subsequent exchange by Ca^2+^ through the reverse mode of the Na^+^/Ca^2+^ exchanger, as demonstrated in pairs of isolated rat cardiomyocytes [425]. In one study, microinjection of extracellular medium into one of the cells to simulate sarcolemmal disruption induced a marked increase in Na^+^ and Ca^2+^ in the adjacent cell and its hypercontracture in less than 30 s, a process that was prevented by the gap junction uncoupler heptanol [437] and the Na^+^/Ca^2+^ exchanger inhibitor KB-R7943 [425].

However, chemical coupling and spreading of injury through gap junctions does not only occur during reperfusion. Studies in an intact rat myocardium provided direct evidence that residual communication through gap junctional channels also exists during the first minutes of ischemia, allowing passage of pathophysiologically relevant molecules [438]. As a consequence, propagation of the cytosolic derangements occurring during ischemia may lead to synchronization of rigor contracture and spreading of cell death [438]. Further confirming this possibility, it has been described that pre-treatment with 1 mmol/L heptanol before the ischemic event reduces infarct size in isolated rabbit hearts [439], although this was not confirmed by other authors at a concentration of 0.5 mmol/L [440]. Similarly, other gap junction uncouplers given during hypoxia were also able to attenuate cardiac injury after reoxygenation in isolated rat hearts [441]. Despite these findings, results obtained with heptanol should be interpreted with caution as this alcohol was suggested to induce an anti-ischemic action, delaying rigor onset and cell-to-cell electrical uncoupling [441].

##### Studies in Transgenic Mice Models

Most of the previous findings were obtained using pharmacological tools that show side effects in other cells targets, including other sarcolemmal channels or the mitochondria [442,443,444,445,446,447]. To further confirm the involvement of gap junctions in spreading of cell injury, transgenic mice models were used. An early study conducted in heterozygous Cx43^+/−^ showed a reduction in infarct size 8 days and 10 weeks after permanent coronary occlusion [448]. However, reductions in healing or fully healed myocardial infarctions in this study might be due to effects of Cx43 deficiency on cardiac remodeling and collagen deposition, but not to acute actions on spreading of cell injury. Accordingly, others were not able to confirm a reduction in infarct size in the same animal model following transient coronary occlusion, 2 h after reperfusion [449,450]. Despite this finding, other animal models support the notion that chemical coupling through gap junctions plays a role in propagation of ischemia/reperfusion injury. Thus, replacement of Cx43 by Cx32 in Cx43KI32 mice was associated with a reduction in hypercontracture, enzyme release and infarct size one hour after reperfusion in isolated mice hearts [394]. Furthermore, ablation of Cx43 expression in Cx43^Cre-ER(T)/fl^ mice after treatment with 4-OHT induced a marked reduction in infarct size as compared with that found in hearts from wild-type animals [294]. Further supporting the involvement of gap junctions in the propagation of injury, it was demonstrated that loss of the CT regulatory domain of Cx43 in mice harboring one Cx43 knock-out allele and one K258stop allele, thus preventing chemical gating of the channels and keeping them opened, led to an increase in infarct size [451]. Furthermore, increasing cell-to-cell coupling by adenovirus-mediated Cx32 gene transfer to wild-type mice also resulted in enhanced infarct size [452].

##### The “Good Samaritan” Effect

Despite the previous evidence, other studies have suggested that GJIC may reduce, instead of increase, the susceptibility of cardiomyocytes to injury. According to this hypothesis, GJIC would dilute the intracellular derangements occurring during the insult into a bigger mass of tissue or would allow transfer of unknown survival factors [453,454]. Thus, the protective effect of ischemic preconditioning has been associated with the transfer of some survival signals during preconditioning cycles, as pre-treatment with heptanol abolished preconditioning protection [455]. Similarly, heptanol abolished protection induced by pre-treatment with δ-opiod agonists [456], whereas the antiarrhythmic effect of ischemic preconditioning was attenuated by previous administration of the gap junction uncoupler carbenoxolone [457]. However, results with heptanol should be considered with caution, at least for the case of ischemic preconditioning, as this alcohol may have anti-ischemic actions that may render preconditioning cycles ineffective [441]. In the same line, however, are some studies using the antiarrhythmic peptides rotigaptide and GAP-134, which are known to improve gap junctional communication, probably through changes in connexin phosphorylation. Both peptides have been shown to reduce chronic infarct size after regional ischemia/reperfusion in anesthetized rats [458] and acute myocardial infarction 2–4 h after reperfusion in dogs [402,403] and pigs [459]. This phenomenon, termed “the Good Samaritan effect”, is not contradictory to the previously discussed spread of injury, as it is conceivable that chemical coupling through gap junctions moves in one or the other direction, depending on the intensity of the insult [84]. In contrast to ischemic preconditioning, however, Cx43 seems not to play a role in postconditioning protection, a protective maneuver consisting of brief cycles of ischemia/reperfusion applied at the time of flow restoration, immediately after the sustained ischemia [460,461].

##### Chemical Coupling through Other Cardiac Connexin Isoforms

Interestingly, chemical coupling through gap junctions is not restricted to Cx43, the main connexin isoform expressed in ventricular cardiomyocytes. Thus, mice with an endothelial-specific deletion of Cx40 were shown to depict a significant increase in myocardial infarct size following 30 min of ischemia and 24 h of reperfusion [462]. Although these findings refer to a different cell type (i.e., endothelial instead of cardiomyocytes), they serve to confirm that chemical coupling through gap junctions is an extended phenomenon beyond cardiomyocytes.

##### Chemical Coupling and Regulation of Cell Growth, Migration and Differentiation

Chemical coupling through gap junctions can also be involved in regulation of cell growth, migration and differentiation, a function that has been mainly explored in tumoral cells [463,464]. In this regard, an inverse relationship between GJIC and tumor cell growth has been described in many cell types, for different tumors and connexins [464,465]. Thus, whereas a reduction in gap junctional coupling by downregulation of Cx43 expression by siRNA has been found to enhance cell growth and migration in human breast cancer cell lines [466], enhanced coupling by connexin overexpression has been shown to act as a tumor suppressor [467]. Gap junctional coupling may exert a control over these functions thanks to the presence of connexin response elements in the promoter region of different genes. Indeed, connexin response elements have been described to modulate gene transcription, including those coding for osteocalcin or collagen Iα1, in osteoblasts in a gap junction-dependent manner [468,469]. Gap junctional communication would allow propagation of second messengers that would activate the ERK/PI3K signaling cascade, resulting in recruitment of the transactivator Sp1 to the connexin response element within the different promoters [469]. Despite this evidence, however, no studies have explored the role of chemical coupling through gap junctions in the control of cell growth in cardiac cells.

Taken together, these data seem to suggest that chemical coupling through gap junctions can play an important role in myocardial ischemia/reperfusion injury. However, it is important to take into account that pharmacological strategies target not only intercelullar channels but also free, unopposed connexin hemichannels. Furthermore, a similar concern can be applied to transgenic mice models. Thus, results obtained under these conditions cannot exclude that free hemichannels are also involved in cell death secondary to coronary artery occlusion, a possibility that will be discussed in the following section.

#### 3.2.3. Gap Junction-Independent Functions of Unopposed Sarcolemmal Hemichannels: Involvement in Paracrine Communication and Dysregulation of Cell Homeostasis

The presence of unopposed hemichannels outside gap junctional plaques was demonstrated using a variety of biochemical and electrophysiological techniques, which allowed establishing that free connexons have a high conductance and permeability. Due to these characteristics, hemichannels are submitted to a strict regulation [86,470], as their uncontrolled opening may result in three main deleterious consequences: loss of cell homeostasis caused by depletion of cytoplasmic metabolites, cell edema due to water influx and plasma membrane depolarization. Indeed, these alterations, which occur following hemichannel opening, have been suggested to be involved in cell injury occurring under some pathological conditions, including ischemia/reperfusion injury.

##### Flux of Intracellular Metabolites through Opened Hemichannels

The function of connexin hemichannels is tightly regulated by the same factors that modulate gap junction gating, including changes in phosphorylation [138] and oxidative stress [213], and by the contractile system [471]. Calcium concentrations also play a major role, with a decrease in calcium’s extracellular levels leading to connexon opening [472,473]. Furthermore, they are also gated by the transmembrane potential, with depolarization increasing their open probability [474], and by intracellular pH [108]. It has been demonstrated that removal of extracellular calcium enhances the Cx43 hemichannel pore diameter from 1.8 to 2.5 nm [130], thus increasing its permeability to a variety of intracellular messengers [475]. Indeed, opening of connexin hemichannels allows release of metabolites such as ATP, NAD^+^, lactate or glutamate [476,477,478,479,480,481], which may have important physiological functions, including paracrine communication between adjacent cells [482]. This may be the case of Ca^2+^ wave propagation.

Calcium waves may propagate by two different mechanisms [432]. The first one involves intracellular diffusion through gap junctions of cytosolic messengers, including IP3 [430,433], as discussed in previous sections. The second mechanism depends on activation of surface membrane receptors by released metabolites, which would, then, initiate a cytosolic signaling cascade in the neighboring cell, resulting in IP3 production and Ca^2+^ release from internal stores. It has been demonstrated that calcium wave propagation in astrocytes and osteocytes depends, at least in part, on ATP release to the extracellular space and its binding to purinergic receptors in neighboring cells [431,478,483,484,485,486]. Connexin hemichannels may constitute the source of ATP, by allowing its extrusion to the extracellular space [477,478,479]. An elegant demonstration of the involvement of Cx43 hemichannels in ATP release and calcium wave propagation in endothelial cells was provided by the finding that Gap26, a connexin mimetic peptide reproducing a sequence located on the first extracellular loop of the protein, was able to block calcium wave propagation independently of changes in gap junctional communication [487]. Furthermore, the release of ATP in individual astrocytes, not connected to other cells, was shown to be abolished by gap junction uncouplers [488]. Although other mechanisms for ATP release may exist [463,475,486], the involvement of connexin hemichannels is, at present, not disputed. Calcium waves are also present in cardiac myocytes [489], although the role played by connexin hemichannels in their propagation has not been properly investigated.

In addition to ATP, Cx43 hemichannels can also mediate bidirectional NAD^+^ fluxes, as demonstrated in murine 3T3 fibroblasts treated with Cx43-antisense oligonucleotides [480], or uptake of extracellular cAMP in HeLa cells expressing this isoform [77]. However, release of these metabolites not only fulfills a physiologic function but they may also have a role under pathological conditions. In this regard, massive release of ATP has been demonstrated to induce apoptosis in chicken lymphocytes, an effect mediated by activation of P2X_7_ purinoceptors and chloride influx [490]. Breakdown of ATP to adenosine might be involved in these effects, at least in some cell types, such as mouse neuroblastoma N1E-115 cells [491]. Similarly, glutamate may trigger excitotoxic injury in the central nervous system due to activation of NMDA receptors, triggering excessive calcium influx, which would be passively followed by chloride and water [492].

##### Calcium Influx and Cell Edema

Release of intracellular mediators is not the only mechanism by which hemichannel opening may influence cell death under pathological conditions. Connexon opening has been shown to compromise ionic homeostasis by allowing calcium influx. Thus, it has been demonstrated that calcium overload induced by extracellular alkalinization in HeLa cells was dependent on the presence of active Cx43 hemichannels [493]. In this regard, calcium overload is a central component of cell death during ischemia/reperfusion injury, at least in the heart, where it is responsible for the appearance of cardiomyocyte hypercontracture, calpain activation and mitochondrial permeability transition pore opening [475,494,495,496]. Furthermore, ion entrance to the cytosol would be passively followed by water influx, leading to cell edema. Thus, it was demonstrated that overexpression of Cx46 in Xenopus oocytes depolarizes and lyses cells within 24 h, effects associated with permeability to Lucifer Yellow, the appearance of voltage-gated currents in non-junctional membranes and water influx [497]. These effects were prevented by increasing the osmotic strength in the extracellular buffer with Ficoll, thus supporting a role for water uptake in cell death [497]. In a similar way, a reversible increase in cell volume was observed in several cell types expressing Cx43 when extracellular calcium was reduced from 1.8 mM to 10 µM, a finding that was not apparent in cells expressing low connexin levels or treated with hemichannel blockers [473]. While volume regulation might be one of the physiological functions of connexin hemichannels, under pathological conditions, an excessive water influx might lead, thus, to cell death.

##### Involvement of Unopposed Hemichannels in Myocardial Ischemia/Reperfusion Injury

Ischemia/reperfusion injury, including that occurring during myocardial infarction, is, as mentioned before, one of the pathological conditions in which opening of connexin hemichannels has been involved. Indeed, ischemia has been demonstrated to favor transient hemichannel opening. Thus, exposure of cortical astrocytes to metabolic inhibition induced permeabilization to Lucifer Yellow and ethidium bromide, effects attenuated by octanol and 18α-glycyrrhetinic acid, and by targeted deletion of Cx43 in transgenic mice [206]. Similarly, HEK293 cells submitted to metabolic inhibition were also shown to develop large non-selective currents and enhanced permeability to calcein, effects blocked by halothane or lanthanum, two known gap junction and hemichannel inhibitors [128]. Importantly, comparable findings were obtained in cardiac cells obtained from adult rabbit hearts [128]. Furthermore, opening of connexons during simulated ischemia has been confirmed in rat neonatal cardiomyocytes, where they increase ATP release [498] and promote cell injury, as suggested by the fact that blockade with the Cx43 mimetic peptide Gap26 was able to improve cell viability [499]. Taken together, these findings may indicate that connexin hemichannels could compromise cell homeostasis during myocardial ischemia and may play a role in arrhythmogenesis and myocardial injury [128,129,500]. This hypothesis was further supported by the finding that treatment with the mimetic peptides Gap26 or Gap27 (Figure 4) was able to reduce infarct size in isolated rat hearts, both when given before or after ischemia [311,501,502]. However, protection against simulated ischemia was not reproduced in isolated rat neonatal cardiomyocytes treated with either Gap26 or 18α-glycyrrhetinic acid [498].

Due to these discrepancies and to the fact that prolonged incubations with Gap26 or Gap27 may also affect gap junction function, in addition to hemichannel opening, further confirmation of the role of hemichannels in ischemia/reperfusion injury was needed. In this regard, promising results have been obtained with the new nonapeptide Gap19, a mimetic peptide derived from the cytoplasmic loop of Cx43 (Figure 4) [143,503]. Gap19 was shown to inhibit Cx43 hemichannel currents without any effect on gap junctional channels or in Cx40 or pannexin hemichannels, both in HeLa cells and in isolated pig ventricular cardiomyocytes [504]. Interestingly, Gap19 prevented hemichannel opening induced by metabolic inhibition, protected against volume overload and cell death in isolated cardiomyocytes and reduced infarct size in an in vivo mice model of transient coronary occlusion [504]. Although this last effect was modest when compared with that obtained with gap junction uncouplers [378,437] or in transgenic mice models [294,394], these results point to a role of Cx43 hemichannels in myocardial infarction. The relative contribution of intercellular gap junctional channels and hemichannels to myocardial ischemia/reperfusion injury deserves, however, further investigation.

Connexin hemichannels may behave, however, as a double-edged sword. While sustained openings may induce toxic effects, either through receptor-mediated mechanisms [487,490,492,505] or by allowing ion and water influx [473,493], transient activation might be involved in protective signaling, including that of ischemic preconditioning, at least in astrocytes and C6 cells [506] and cultured neurons [507]. These protective effects have been proposed to be due to ATP efflux and accumulation of adenosine, a known protective agent [506,507]. However, the role of connexin hemichannels in cardiac preconditioning protection is controversial and probably negligible, as others authors have suggested that this protective maneuver might reduce opening of hemichannels and thus reduce their deleterious consequences on the cytoplasmic depletion of metabolites and on calcium and water overload [508]. Moreover, a peak of ATP release through opened hemichannels occurs only after more than 80 min of ischemia in rat neonatal cardiomyocytes, as compared with the brief duration of preconditioning cycles [498].

#### 3.2.4. Involvement of Sarcolemmal Connexins in Long-Distance Communication through Tunneling Nanotubes and Extracellular Vesicles

Connexins have been described to also be expressed in tunneling nanotubes (TNTs), highly dynamic membrane protrusions that extend for up to 100 µm (diameter between 50 and 700 nm), and that allow transfer of different cytoplasmic components, including proteins, miRNAs, intracellular vesicles or even organelles, between two connected cells [509,510]. TNTs, which are characterized by the presence of F-actin and sometimes microtubules, have been observed in many cell types, including cardiac cells [509,510]. Thus, thin-membrane nanotubular structures containing actin and microtubules have been described connecting cultured neonatal rat ventricular cardiomyocytes and cardiofibroblasts [511] or mesenchymal stem cells [512], but also within adult mouse cardiac tissue [511], where they may mediate the transfer of functional mitochondria or allow calcium propagation. Importantly, TNT formation is enhanced by ischemia, both between cultured cardiomyocytes and fibroblasts and in rat and human cardiac tissues [513], and TNTs have been proposed to impact on arrhythmogenesis, fibrosis or injury resistance [510]. Although not all TNTs are end-to-end connected through connexins [509], the presence of connexons may facilitate docking of TNTs to the target cell [514,515] and, in this way, may be involved in long-distance intercellular electrical and chemical coupling [509,515,516,517]. However, few studies have addressed the role of TNTs in cardiac pathophysiology.

Extracellular vesicles are membrane-formed structures, ubiquitously found throughout the organism, that are released into the bloodstream by a variety of cells to elicit a response in distant organs or tissues. They can be divided, according to their size and subcellular origin, into exosomes, having a diameter ranging between 50 and 200 nm, which are secreted after fusion of multivesicular bodies to the cell membrane, and microvesicles (100–1000 nm), which are formed by outward budding of the plasma membrane (Figure 3) [509,518]. Extracellular vesicles may exert direct effects in cardiac tissues and may be released by the heart itself. Thus, exosomes derived from cardiac fibroblasts have been described to induce cardiomyocyte hypertrophy, an effect mediated by miR-21_3p [519]. Furthermore, exosomes derived from different cell types, even those isolated from plasma from human volunteers, have been shown to exert a cardioprotective action against cardiac ischemia/reperfusion injury both in vivo and in isolated heart models [520,521,522], and they might be involved in the cardioprotective effect of remote ischemic conditioning [523,524]. Circulating extracellular vesicles, enriched in miRNAs, are released from the myocardium during ischemia/reperfusion and may constitute an early biomarker of cardiac injury [525], and their content can be influenced by ischemic preconditioning [526]. Importantly, recent evidence indicates that the collection of proteins expressed at the membrane of extracellular vesicles may determine their tropism and modulate how their content is released into the receptor cell [509,527]. In this regard, expression of several connexins, including Cx43, Cx45 and Cx32, has been demonstrated in extracellular vesicles of different origins [509], including those derived from cardiac cell lines [528]. Accordingly, Cx43 was shown to be present as hexameric hemichannels in exosomes derived from H9c2 cardiomyoblasts, and it has been proposed that it may modulate the interaction and transfer of exosome intraluminal content to receptor cells [528]. Expression of Cx43 in cardiac-derived extracellular vesicles has been described to be reduced after myocardial infarction [529]. However, further research efforts are needed to fully understand the role of Cx43 in cardiac-derived extracellular vesicles and its importance for tissue function.

#### 3.2.5. Mitochondrial Connexins

##### Presence of Cx43 at Cardiomyocyte Mitochondria

The first pieces of evidence on the presence of Cx43 at the mitochondria were obtained in homocysteine-treated human umbilical vein endothelial (HUVEC) cells [530]. Thus, whereas confocal microscopy studies conducted in control cells confirmed the classical localization of Cx43 at the plasma membrane, treatment with homocysteine enhanced total expression of the protein, a finding not linked with changes in gap junctional communication, and induced its intracellular redistribution to the mitochondrial compartment [530]. Furthermore, immunoblotting studies in isolated mitochondria confirmed overexpression of Cx43 at this organelle in homocysteine-treated HUVEC cells [530].

Later studies, intended to explore the involvement of Cx43 in the mechanisms of myocardial preconditioning protection, were also able to identify the protein in mitochondria from cardiomyocytes [23,24,25,531]. Western blot analysis of non-contaminated mitochondrial preparations allowed demonstrating the presence of Cx43 in this organelle in several species, including rat, mouse, pig and human [23]. These findings were corroborated by FACS sorting and immuno-electron and confocal microscopy [531], and by proteomic analysis [531]. Furthermore, the location of Cx43 at the mitochondria was later confirmed by other authors, not only in endothelial cells [530] or in cardiac preparations [442,532,533,534,535] but also in the brain [536,537], astrocytes [538] and bone marrow stem cells [539]. Subfractionation studies allowed demonstrating, later, that Cx43 is mainly located at the inner mitochondrial membrane, where it represents about 4% of total cardiomyocyte Cx43 [24]. Although Cx43 lacks a mitochondrial entry pre-sequence at the N-terminal part of the molecule [540], which is normally enriched in serines and threonines, other mechanisms of import exist requiring the activity of cytosolic chaperones [541]. Indeed, this isoform has been shown to be translocated to this location by the regular mitochondrial protein import machinery, including the TOM/TIM system and Hsp90 [24]. In fact, Cx43 co-immunoprecipitates with Tom20, one of the components of the multiproteic complex TOM, and with Hsp90, and its translocation to the organelle is attenuated by geldanamycin, an Hsp90 inhibitor [24]. Similarly, its location at the inner mitochondrial membrane has also been demonstrated in mouse bone marrow stem cell antigen-1^+^ (Sca1^+^) cells, where its expression was increased after insulin-like growth factor-1 (IGF-1) treatment [539], and in rat brain mitochondria [536]. However, some discrepancies exist in this regard, as other authors have suggested, using subfractionation methods, that Cx43 is located at the outer mitochondrial membrane in mitochondria isolated from rat adult ventricles [533]. Reasons for these discrepancies are unknown. Importantly, Cx43 is almost exclusively located at subsarcolemmal, but not interfibrillar, mitochondria, with its CT directed towards the intermembrane space (Figure 3 and Figure 6) [542]. Furthermore, cross-linking studies have demonstrated the presence of Cx43 as hexamers or hemichannels in mitochondrial membranes [531]. In addition to the TOM/TIM system, it is not possible to exclude that Cx43 may use other delivery systems to reach the mitochondria. In this sense, delivery of Cx43 to the mitochondria might also occur through annular gap junctions, as immunocytochemical, super-resolution and transmembrane electron microscopy studies have demonstrated a surprisingly greater frequency of associations of annular gap junctions with this organelle as compared with lysosomes [543].

##### Functions of Mitochondrial Cx43

Functions of mitochondrial Cx43 are largely unknown. Mitochondrial K^+^ influx was reduced in digitonin-permeabilized cardiomyocytes from Cx43KI32 mice, lacking Cx43, as compared with those from wild-type animals, whereas treatment with 18α-glycyrrhetinic acid inhibited this influx in cells from wild-type mice but not in those from Cx43KI32 animals [531]. These initial studies were later extended to subsarcolemmal mitochondria isolated from Cx43^Cre-ER(T)/fl^ mice treated with 4-OHT to induce a marked ablation of the protein, or from wild-type mice treated with the mimetic peptide Gap19 [544]. Taken together, these findings allowed suggesting that mitochondrial Cx43 impacts on K^+^ fluxes in this organelle [531]. In addition, mitochondrial Cx43 might also contribute to mitochondrial Ca^2+^ homeostasis, thus being involved in mitochondrial permeability transition pore opening and cell death [545]. Furthermore, subsarcolemmal mitochondria from rat hearts depicted a reduction in ADP-stimulated complex I respiration and ATP generation after treatment with 18α-glycyrrhetinic acid or Gap27 [546]. Similar results were obtained in conditional Cx43^Cre-ER(T)/fl^ mice treated with 4-OHT, which showed a decrease in ADP-stimulated complex I respiration, but not in complex II respiration [546]. These data may indicate, thus, that mitochondrial Cx43 modulates complex I respiration and oxygen consumption [546].

##### Involvement in Preconditioning Protection

Several studies have demonstrated that animal models devoid of Cx43 cannot be preconditioned. This was the case of a Cx43 null mice model, which was shown to be insensitive to hypoxic preconditioning when submitted to occlusion of the middle cerebral artery [506]. At the cardiac level, a reduction in Cx43 levels in heterozygous Cx43^+/−^ mice abolished preconditioning protection, whereas protection remained in wild-type animals [449,450]. Taken together, these findings indicate that Cx43 is essential for preconditioning protection. Furthermore, ischemic or pharmacological preconditioning has been shown to be associated with enhanced Cx43 levels, either in the myocardium [547] or in glial C6 cells [506], with preservation of Cx43 phosphorylation during the sustained ischemic event [456,508,548,549], with a reduction in gap junction permeability [548,549] and with redistribution of the protein from the intercalated discs to the lateral membranes [303]. These last findings would, hypothetically, indicate that preconditioning favors gap junction closure during ischemia/reperfusion, thus preventing diffusion of death factors between neighboring cells and increasing cell survival.

However, detailed electrophysiological studies, conducted in isolated rat hearts or in situ pig hearts, showed that electrical uncoupling during the ischemic phase was not delayed by ischemic preconditioning [423]. Furthermore, no differences in tissue resistance normalization during reperfusion were observed between control and preconditioned hearts [423]. These results strongly suggested that protection by ischemic preconditioning was not secondary to a reduction in gap junction-mediated propagation of injury. Supporting this possibility, it was found that freshly isolated rabbit or mice cardiomyocytes, which are not connected through gap junctions, could still be preconditioned [550,551,552]. Similar results were obtained in other tissues. Overexpression of Cx43 in astrocytes was protective against cell death induced by several insults, and this effect was preserved in cells plated at low densities, treated with gap junction uncouplers, or in cells expressing a mutation rendering Cx43 channels non-functional [553]. In addition, Cx43-deficient C6 glial cells responded to preconditioning only after exogenous expresson of the protein, whereas protection was attenuated by siRNA [506]. Importantly, whereas preconditioning was effective in isolated cardiomyocytes from wild-type mice, those from heterozygous Cx43^+/−^ animals were not protected by the manoeuver [551]. These data would indicate, thus, that the essential role of Cx43 in preconditioning protection is mostly independent of gap junctional communication. However, some role of gap junctions cannot be completely discarded as protection is usually higher in whole hearts than in isolated cells [508].

While the contribution of free, unopposed Cx43 hemichannels to preconditioning protection during a sustained ischemic episode remains to be elucidated [25], that of mitochondrial Cx43 is becoming more apparent. Such possibility was supported by the fact that preconditioning cycles enhance mitochondrial Cx43 levels [23]. Moreover, diazoxide, a drug inducing protection through activation of mitoK_ATP_ channels, enhanced ROS production in cardiomyocytes from wild-type animals, but not in those isolated from Cx43^+/−^ animals [552]. This functional defect of Cx43-deficient cardiomyocytes was specific for diazoxide, as other drugs inducing ROS through different mechanisms were still effective in these cells [552]. Moreover, effects of diazoxide on ROS production correlated with a lack of efficacy of this pharmacological preconditioning in hearts from Cx43-deficient animals [552]. As mitochondrial ROS production plays a key role in preconditioning protection [554], these data are suggestive of an involvement of mitochondrial Cx43 in mitochondrial ROS production and in preconditioning protection. Indeed, protection by diazoxide was abolished when Cx43 translocation to the mitochondria was reduced by Hsp90 inhibition with geldanamycin [24]. Furthermore, preconditioning with diazoxide was also abolished in isolated hearts from heterozygous Cx43KI32 mice [394] and Cx43^Cre(ER)T/fl^ animals [294], whereas hearts from homozygous Cx43KI32 mice and Cx43^Cre(ER)T/fl^ animals treated with 4-OHT were neither protected by pharmacological nor ischemic preconditioning [294,394]. Taken together, these findings support an essential role for mitochondrial Cx43 in preconditioning protection, especially that dependent on ROS production by this organelle, as is the case of diazoxide [25]. This is further supported by the finding that mitochondrial Cx43 physically interacts with the Kir6.1 subunit of mitoK_ATP_ channels [276]. It has been hypothetized that the involvement of mitochondrial Cx43 in preconditioning protection can be explained, at least in part, by changes in the degree of S-nitrosation of the protein. In fact, S-nitrosation of mitochondrial Cx43 is known to regulate mitochondrial function, enhancing mitochondrial permeability, especially for K^+^, and ROS formation [555]. In this regard, it has been hypothesized that mitochondrial Cx43 levels may control the exact isoform of the nitric oxide synthase (NOS) expressed at the organelle [209]. Thus, mitochondria from Cx43^Cre-ER(T)/fl^ mice depicted a switch from the predominant nNOS observed in wild-type animals to the iNOS isoform in Cx43-deficient mice [209]. This would cause a reduction in mitochondrial NO formation and in protein S-nitrosation, which, in turn, would attenuate mitochondrial K^+^ influx and ROS production upon external stimuli such as ischemic preconditioning [209].

Mitochondrial Cx43 might also exert a protective action against calcium-induced mitochondrial permeability transition pore opening [536]. In this way, it might be involved in the protective effect of fibroblast growth factor 2 (FGF-2) [556], which is dependent on Cx43 phosphorylation at Ser262 [310]. However, and in contrast to ischemic preconditioning, Cx43, including mitochondrial Cx43, is not involved in postconditioning protection [460,461].

##### Involvement in Chemotherapy-Induced Cardiotoxicity

It has been suggested that mitochondrial Cx43 also exerts an important protective role against cardiotoxicity induced by antitumor drugs. This is the case of doxorubicin, an anthracycline-derived chemotherapeutic agent used against a wide range of malignant tumors. Although the mechanisms of doxorubicin cardiotoxicity are complex and varied, mitochondrial dysfunction is among the most prominent [557]. In this regard, it has been shown that pharmacological inhibition of Hsp90 with radicicol, a treatment reducing mitochondrial Cx43 expression in H9c2 cardiomyoblasts, increased cardiotoxicity to doxorubicin, as determined by an enhancement in doxorubicin-induced ROS production, in mitochondrial calcium overload and in cytochrome C release [535]. Additionally, an increase in nitrosative stress was also observed after radicicol treatment [558]. Similar findings have been obtained with trastuzumab, the gold standard in the treatment of HER2+ breast cancer [559]. Furthermore, doxorubicin alters Ca^2+^ homeostasis in cardiomyocytes from mice treated with doxorubicin, and this effect is associated with compensatory changes, including a reduction in total Cx43 expression, with enhanced translocation of the protein to the mitochondrial compartment and with increased phosphorylation at Ser368 [560]. Interestingly, diazoxide attenuates doxorubicin-induced cardiotoxicity in mice and this effect is associated with an increase in Cx43 expression both at the sarcolemma and within the mitochondria [561]. Further research, including experiments in Cx43-deficient mice models, is, in any case, needed, in order to confirm the validity of these findings, and to fully elucidate the role of mitochondrial Cx43 in cardioprotection.

##### Other Mitochondrial Connexins

Interestingly, Cx43 is not the only connexin isoform that has been described in the mitochondria. Western blot and immunofluorescence studies allowed establishing that Cx40 is also located at the mitochondria in mice coronary endothelial cells [562]. Cx40 knock-out mice exhibited reduced resting and stimulated mitochondrial Ca^2+^ concentrations and ROS production, suggesting that it may play a role in mitochondrial Ca^2+^ homeostasis [562]. Whether these findings extend to other connexin isoforms, beyond Cx43 and Cx40, is currently unknown.

#### 3.2.6. Nuclear Connexins

The presence of Cx43 at the nucleus of tumoral cells was described by confocal and Western blot analyses of transformed rat liver epithelial cells more than 20 years ago [563]. These initial findings were later confirmed in mouse and human lung carcinoma cells [564], in Cx43-overexpressing glioblastoma cell lines [565], in a panel of human gliomas [566], in human colorectal tumors [567] and in mice chondrocytes [568], among many others. Additionally, the location of Cx43 at the nucleus was shown to dynamically change during the mitotic process in lung A549 adenocarcinoma cells [569]. The nuclear location of Cx43 might be explained by the existence of a putative nuclear-targeting sequence encoded within its CT domain [570,571], and its translocation might depend on phosphorylation at Ser255, as it has been proposed in human endometrial cells [572]. Furthermore, Wnt signaling has been suggested to regulate translocation of Cx43 from the cytosol to the nucleus in PC3 human prostate cancer cells, where it may constitute a cotranscription factor of β-catenin [573]. Importantly, nuclear Cx43 expression may predict a worse overall survival, at least in lung cancers [574].

**Figure 7:**
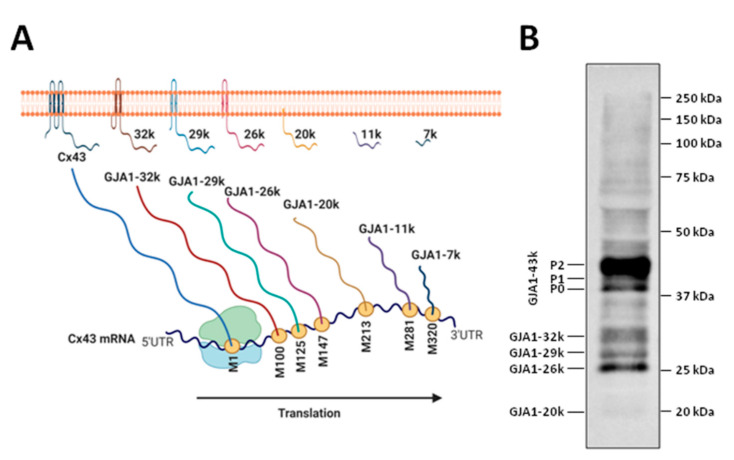
(**A**) Schematic representation of the GJA1 transcript encoding Cx43 and up to 6 putative N-terminally truncated forms. Suggested internal translation initiation sites corresponding to starting AUG codons, encoding for methionines, are shown as yellow circles. Created with Biorender.com (accessed on 15 April 2021). Modified from [8,577]. (**B**) Representative Western blot showing expression of full-length Cx43 (P2, P1 and P0 phosphorylation states), together with the putative N-terminally truncated isoforms GJA1-32k, GJA1-29k, GJA1-26k and GJA1-20k, in tissue extracts obtained from a normoxic isolated rat heart.

Transfection with a vector encoding the full-length protein in glioblastoma cell lines suggested overexpression of full-length Cx43 at the nucleus [565]. However, detection of Cx43 at the cell nucleus has usually been conducted with antibodies raised against its carboxyterminus, and, thus, they were not able to differentiate between the full-length protein and truncated fragments derived from its CT domain. In fact, active translation of up to six different N-terminally truncated Cx43 isoforms, including a 20 kDa fragment (GJA1-20k), has been confirmed by different authors in a number of cell types [575,576,577]. Interestingly, expression of at least four of these N-terminally truncated Cx43 isoforms, GJA1-32k, GJA1-29k, GJA1-26k and GJA1-20k (Figure 7), has also been described in human cardiac cells, with the last isoform listed being the predominant one [578]. Furthermore, a peptide migrating at about 25 kDa was present in isolated rat hearts (Figure 7), even in mitochondria, and was upregulated after ischemia [464]. Truncated isoforms are thought to be formed by internal translation initiation within the Cx43 mRNA, in a process regulated by signaling pathways including mTOR and Mnk1/2 kinases [575,576,578]. GJA1-20k may function as a cytosolic chaperone, regulating the traffic of full-length Cx43 to the plasma membrane [578]. Furthermore, it may also promote mitochondrial transport through microtubules [579]. Importantly, at least part of these N-terminally truncated isoforms can be expressed at the nucleus, as demonstrated in the case of GJA1-20k, in a rat glioma cell line [580]. In addition, transfection of cardiomyocytes with the CT tail of Cx43 (residues 243–382) resulted not only in a wide cytoplasmic expression of the protein but also in its location being at the cell nuclei, as assessed both by immunofluorescence and by Western blot analysis of subcellular fractions [22].

Functions of nuclear Cx43 are largely unknown and have been mostly studied in tumoral cells. In addition to the previously discussed effects of chemical coupling through gap junctions in the control of cell growth, migration and differentiation, Cx43 may also modulate these functions by gap junction-independent mechanisms [463,464,581,582,583]. This was first suggested after transfection of HeLa cells with vectors encoding for Cx26, Cx40 and Cx43 [584]. In this study, transfection with all three connexin isoforms allowed the establishment of functional gap junctions, but only cells transfected with Cx26 had a strong negative effect on cell growth, and there was no clear correlationship between GJIC and tumorigenicity [584]. A lack of correlation between cell coupling and growth was also observed in 3T2 A31 fibroblasts overexpressing several Cx43 mutants [585]. Furthermore, culture at low densities or treatment with the gap junction uncoupler heptanol did not modify the growth-regulatory effects of these mutants [585]. In a similar line, transfection of human brain glioblastoma cells with Cx43 reduced proliferation in monolayer cultures and athymic nude mice, effects that were not associated with the establishment of gap junctional communication [565], and retroviral delivery of Cx43 and Cx26 to human breast tumor cells resulted in a reduction in tumor growth, with no detectable gap junctions nor rescue of intercellular communication [570]. In addition, point mutations in the second extracellular region of Cx43, which induced loss of the protein at the plasma membrane, did not modify its ability to suppress cell growth in HeLa cells [586]. Interestingly, transfection of either the full-length protein or its CT domain was equally effective, reducing cell growth and proliferation in Neuro2a cells [585], a finding also obtained in HeLa cells [22]. The CT domain of chicken Cx45.6 has also been suggested to be of importance in lens epithelia–fiber cell differentiation [581,587]. These last findings may indicate that it is not the full-length Cx43 but instead its CT domain which exerts these effects on cell growth, migration and differentiation.

The effects of connexins on cell growth and differentiation may be due to actions on DNA synthesis and regulation of transcription, processes in which nuclear connexins migth be involved [463,464]. In this regard, overexpression of wild-type Cx43 in rat neonatal cardiomyocytes has been shown to reduce DNA synthesis irrespective of the presence of cell-to-cell contacts [588]. In fact, gene array analysis has allowed demonstrating that some isoforms, such as Cx26, are able to regulate the expression of angiogenesis-related genes in human breast tumor cells, both in a gap junction-dependent and independent manner [589], whereas Cx43 deficiency has been associated with multiple transcriptional changes in several tissues [590], including the heart [591]. Importantly, Kotini and coworkers [592] nicely demonstrated that the GJA1-20k isoform may, in fact, act as a transcriptional regulator at the cell nucleus, through a mechanism that is conserved between amphibian and mammalian cells. Basic transcription factor-3 would drive translocation of the Cx43 CT to the nucleus, where it would form a complex with PolII [592]. This complex would, then, bind to the N-cadherin promoter regulating its transcription [592]. In addition, it has been recently shown that GJA1-11k, the 11 kDa alternatively translated isoform of Cx43, preferentially localizes to the nucleus of HEK293FT cells, where it may suppress cell cycle progression, in a gap junction-independent manner [593].

Nuclear Cx43, either as the full-length protein or as its CT domain, may help to explain the effects of Cx43 deficiency on cardiac remodeling and collagen deposition. Previous studies demonstrated that a reduction in Cx43 expression in Cx43^Cre-ER(T)/fl^ mice, having about 50% of the normal Cx43 expression, was associated with an exaggerated fibrotic response after chronic exposure to angiotensin II (AngII) as compared with that occurring in wild-type animals (Cx43^fl/fl^) [594,595]. However, this effect was not evident in heterozygous Cx43^+/−^ animals, having a similar degree of Cx43 deficiency, thus suggesting that this abnormal interstitial collagen deposition was independent of Cx43 expression [595]. On the contrary, a marked Cx43 deficiency, below 5% of the normal Cx43 expression, in Cx43^Cre-ER(T)/fl^ mice injected with 4-OHT, reduced collagen deposition after AngII treatment, as compared with that occurring in their corresponding controls, the AngII-treated, vehicle-injected Cx43^Cre-ER(T)/fl^ mice [595]. This protective effect was associated with enhanced MMP-9 activity and an increased inflammatory reaction in cardiac samples, and reduced fibroblast differentiation capacity, as assessed by α-smooth muscle actin and SM22α expression, in isolated cells [595]. A similar protective action of Cx43 deficiency against collagen deposition, scar formation and cardiac remodeling has also been demonstrated after transient coronary occlusion in the same animal model [596], and in heterozygous Cx43^+/−^ mice, submitted to permanent coronary artery ligation [448,597]. Permanent ligation in the last two studies avoided the acute effects of Cx43 deficiency on infarct size [294,394], a possible confounding factor when analyzing scar size, whereas 4-OHT treatment in the first of these studies [596] was delayed 24 h after coronary occlusion with the same purpose. Similar to these studies, treatment with the CT mimetic peptide αCT1 was shown to attenuate cardiac remodeling and scar area in cryoinjured hearts [598,599]. Whether the effects of Cx43 deficiency on cardiac fibrosis are related to changes in fibroblast function only or whether cardiomyocytes are also involved is currently unknown. Downregulation of Cx43 has been shown to improve wound healing also in other tissues, including the skin and cornea [598,600,601,602,603,604,605], even in humans [606]. Although the exact mechanisms for the regulatory action of Cx43 in the fibrotic response are not completely understood, considering the described effects of the N-terminally truncated Cx43 isoforms in the control of gene expression [592,593], it is tempting to speculate that modulation of gene transcription may play a key role. However, we cannot exclude that these effects are dependent on gap junctional communication, as collagen Iα1 gene transcription has been shown to be modulated in some cell types in a gap junction-dependent manner [468,469]. In addition, these effects can also be related to regulation of other transcription factors, as we have discussed before for β-catenin or β-arrestin [268,273].

## 4. Concluding Remarks

Gap junction-dependent functions of cardiac connexins are key determinants of cardiac pathophysiology. GJIC mediates electrical coupling, allowing impulse propagation between neighboring cardiomyocytes, and its disruption may lead to atrial or ventricular arrhythmias. In addition, GJIC is also involved in chemical coupling, which allows the transfer of cytosolic signals between connected cells, and may be important in propagation of ischemia/reperfusion injury during myocardial infarction. However, in addition to the role connexins play in cell-to-cell communication, mounting evidence demonstrates that connexins have multiple additional functions. Connexins modulate cell growth and differentiation, both in a gap junction-dependent and in a gap junction-independent manner, with the last involving regulation of transcription at the cell nucleus, either by the full-length protein or by N-terminally truncated fragments derived from the CT domain. Furthermore, the non-canonical roles of connexins include those of unopposed hemichannels, whose opening might be involved in paracrine signaling, but also in loss of cell homeostasis and intracellular edema in some pathologies, including myocardial infarction. Mitochondrial connexins may control some steps of mitochondrial respiration and ROS production and may constitute a key player in preconditioning protection and chemotherapy cardiotoxicity. Further studies are required, in any case, to fully unveil the importance of these emerging processes in cardiac physiology and disease.

## Data Availability

Not applicable.
